# Engineering Bacteria to Search for Specific Concentrations of Molecules by a Systematic Synthetic Biology Design Method

**DOI:** 10.1371/journal.pone.0152146

**Published:** 2016-04-20

**Authors:** Shin-Ming Tien, Chih-Yuan Hsu, Bor-Sen Chen

**Affiliations:** Lab of Control and Systems Biology, Department of Electrical Engineering, National Tsing Hua University, Hsinchu, Taiwan; Niels Bohr Institute, DENMARK

## Abstract

Bacteria navigate environments full of various chemicals to seek favorable places for survival by controlling the flagella’s rotation using a complicated signal transduction pathway. By influencing the pathway, bacteria can be engineered to search for specific molecules, which has great potential for application to biomedicine and bioremediation. In this study, genetic circuits were constructed to make bacteria search for a specific molecule at particular concentrations in their environment through a synthetic biology method. In addition, by replacing the “brake component” in the synthetic circuit with some specific sensitivities, the bacteria can be engineered to locate areas containing specific concentrations of the molecule. Measured by the swarm assay qualitatively and microfluidic techniques quantitatively, the characteristics of each “brake component” were identified and represented by a mathematical model. Furthermore, we established another mathematical model to anticipate the characteristics of the “brake component”. Based on this model, an abundant component library can be established to provide adequate component selection for different searching conditions without identifying all components individually. Finally, a systematic design procedure was proposed. Following this systematic procedure, one can design a genetic circuit for bacteria to rapidly search for and locate different concentrations of particular molecules by selecting the most adequate “brake component” in the library. Moreover, following simple procedures, one can also establish an exclusive component library suitable for other cultivated environments, promoter systems, or bacterial strains.

## Introduction

Bacteria navigate to suitable living environments with the help of flagellum propulsion controlled by a sophisticated signal sensing and transduction pathway [1, 2]. Employing techniques from synthetic biology enables scientists to utilize such signal transduction pathways to endow bacteria with novel moving behavior in response to specific stimuli. Based on this concept, we are able to make bacteria automatically search for and locate a particular environment to perform various missions. For example, bacteria could be engineered to seek pollutants in the environment and destroy them [3]. Alternatively, bacteria could also be designed to locate diseased tissue, thus can be employed to synthesize therapeutics or invade cancer cells [4]. It is the searching mechanism that may make pollutant dissolution or drug delivery much more specific and efficient. Here, we use a synthetic biology-based method to create bacterial populations, which can rapidly search for and locate different concentrations of particular molecules.

*Escherichia coli* is chosen to perform the genetic circuits we designed in this study. Like many bacteria, *E*. *coli* swim randomly and switch between two states: smooth runs and tumbles, which usually cause a change in direction. Smooth runs are maintained through counterclockwise flagellum rotation until one or more flagellum rotates clockwise and makes the *E*. *coli* tumble. The switching frequency between the two states is influenced by the chemotactic system. As *E*. *coli* move toward gradients of favorable molecules for growth, the frequency of tumbling will decline as bacteria approach the source. Conversely, if *E*. *coli* deviate from gradients of favorable molecules, tumbling frequency increases to improve the chances bacteria find a favorable environment.

*E*. *coli* detect chemotactic molecules, such as amino acids and sugars, by six transmembrane chemoreceptor proteins. The chemoreceptors control activity downstream of the CheA protein, which autophosphorylates the CheY protein. Phosphorylated CheY protein (CheY-P) binds to the flagellar switch protein FliM and causes the flagellum to rotate clockwise, which results in tumbling of the bacteria. A phosphatase protein, CheZ, is able to dephosphorylate CheY-P, restoring the flagellum to a counterclockwise rotation and enabling the bacteria resume smooth swimming [1, 2, 5]. When binding chemotactic molecules to chemoreceptors, the activity of CheA protein is squelched and thereby CheY protein predominates. In this form, CheY protein fails to interact with FliM protein and causes the flagellum to rotate counterclockwise, which subjects the bacteria to smooth swimming. The above-mentioned signal transduction pathway decides how *E*. *coli* respond when facing chemical signals in the environment. Therefore, interfering with these transduction pathways could provide an effective method for controlling *E*. *coli* movement behaviors.

In recent years, some groups have succeeded in reprogramming bacteria to recognize and trace specific chemicals through protein engineering, RNA engineering, and synthetic biology [6]. Goulian et al. reengineered a wild type Tar receptor, which responds to aspartate, by directed evolution and enabled the Tar receptor to respond differently to phenylacetic acid (PAA) [7, 8]. By transforming a gene encoding penicillin acylase, an enzyme hydrolyzes phenylacetylglycine (PAG) to PAA, enabling *E*. *coli* to ascend PAG gradients. The advantage of direct reprogramming of chemoreceptor proteins is that preserving the sophisticated chemotactic transduction pathway retains the essential merits of the chemotactic system, such as quick response times and adaptions. However, the disadvantage of protein engineering is that only minor modifications can be made based on the existing protein structure. When handling chemicals with extensive changes in structure compared to the native chemotactic molecules, an applicable ligand specificity is hard to achieve.

Gallivan et al. chose an RNA engineering approach [9], using a strain lacking the CheZ gene. Absence of CheZ allows CheY-P to accumulate, which in turn causes the bacteria to keep tumbling rather than swim effectively. The authors coupled a designed riboswitch to respond to theophylline with a ribosome binding site (RBS) and a CheZ encoding gene. Without theophylline, the three-dimensional structure of the riboswitch will shelter the RBS and prevent transcription of the downstream CheZ gene. Once the riboswitch combines with theophylline, the structural transformation will open the RBS to ribosome binding so that the following transcription of CheZ will restore mobility to the bacteria. That is, under this design, the engineered bacteria are able to distribute freely over the region containing theophylline unless they move away from theophylline, which constrains their moving ability. The advantage of this approach is that the components that recognize a specific molecule are RNA aptamers that can be selected *in vitro*. Thus, the design of the aptamer is no longer limited by the scaffold of the receptor protein and can be used to detect new molecules. However, the disadvantage is that this approach relies on CheZ protein production after activation, rather than direct signal transduction by the modified receptor protein. Therefore, response time will be longer compared to the former protein engineering method. Furthermore, only if the molecules of interest pass through the cell membrane will the riboswitch be activated. That is, the sensitivity will be reduced in response to detecting molecules, when part of them may fail to penetrate the membrane.

Cirino et al. studied communication-induced mobility on bacteria by a synthetic biology method [10]. By giving different synthetic circuits, the authors created two *E*. *coli* populations, sender cells and receiver cells, such that the former, which constitutively produces acyl homoserine lactone (AHL), is able to restore the moving ability of the latter. The mechanism is that receiver cells with a MotB gene knockout lose their flagellum rotating ability. When receiver cells are close to sender cells that release AHL, the AHL molecule will initiate the lux system, a quorum sensing system from *Vibrio fischeri*, in the receiver cell's synthetic circuit. Initiating the lux system leads to expression of the MotB gene located behind the lux promoter in the synthetic circuits and thus restores the receiver cells' moving ability. The advantage of using synthetic biology is the increasing homogeneous or heterogeneous detection systems that can be chosen as this field evolves, such as the fdhF promoter which responds to hypoxia in *E*. *coli* [4] and the PpbrA promoter which responds to lead in *Ralstonia metallidurans* [11], to name a few. Similar to RNA engineering approaches, the disadvantage of synthetic biology is its long response time.

In this study, a systematic synthetic biology-based method is designed to achieve the searching function. A CheY double mutant D13K Y106W (CheY**) [12, 13], which is resistant to phosphorylation and can convert into its active conformation to mimic phosphorylated CheY, is chosen in this method to continuously induce tumbling. CheY** production is controlled by the lux quorum sensing system, which will be activated as AHL is detected. Therefore, the *E*. *coli* population will disperse throughout the whole area until they run into the region containing AHL and start tumbling instead.

With previous methods [9, 10], bacteria were deprived of their moving ability until specific molecules were detected, adopting a stop-then-trace strategy. However, if the molecules of interest disperse in the area away from the bacteria at the beginning, the bacteria rarely arrive at the designated area. In this situation, the searching effect can be achieved only if the bacteria have saturated the environment, which requires a considerable amount of bacteria. On the contrary, the synthetic circuits in our design are based on a search-then-locate strategy to solve this searching problem. The ability to explore environmental molecules enables the bacteria to actively and efficiently search for specific molecules. This may reduce the amount of bacteria required for searching. In addition, our design has two additional advantages. First, the method depends on producing CheY** after stimulation. CheY** is able to directly deprive bacteria of movement ability. That is, the method can be applied to most *E*. *coli* strains without modification, whereas some genes, such as CheZ or MotB, must be knocked out in advance under other methods [9, 10]. Second, *E*. *coli* strains can be modified to meet specific requirements without losing the synthetic circuits’ function. For instance, CheY or CheA genes can be knocked out to enable smooth movement. The knocked out genes also block the chemotaxis transduction pathway, which provides a chemotaxis-free experimental environment, neglecting the influence of chemotactic molecules. Additionally, the mediums for measuring bacterial movement are no longer limited to chemotactic molecule-free ones. These modifications cannot be adopted by previous synthetic biology methods, where the lack of CheA or CheY causes *E*. *coli* to fail to tumble in absence of stimulation, which breaks the fundamental stop-then-trace strategy. However, the potential drawback of our design is that bacteria may stop around the source of the molecule instead of invading it precisely owing to the over sensitivity of the circuits.

To solve this problem, we improved our circuit design through several modifications, such as altering the order of genes in the pLux operon, replacing RBSs by weaker ones, inserting a terminator in front of the RBS, and choosing CheY with different mutant levels to design a library of “brake components” with various braking strengths. Putting different “brake components” behind the promoter enables the circuits to respond to molecules of interest with corresponding sensitivities. That is, different “brake components” can be chosen to handle different concentrations of molecules. Stronger components are chosen to construct more sensitive circuits when finding molecules with low concentration. Weaker components are chosen to deal with molecules with high concentrations in order to prevent *E*. *coli* from being excessively sensitive, which makes them stop too far away from the molecular source.

To verify and define how the brake components influence mobility requires applying approaches to measure diffusion rate of *E*. *coli* populations. In this study, two tools, swimming plate assays and microfluidic devices, were used to observe bacteria mobility. Swimming plate assays can simply show how *E*. *coli* populations respond to chemicals by inoculating the population on a semisolid agar plate. While the bacteria population diffuses outward and forms concentric bands, molecules are dropped near the bacteria population to simulate a condition representing a scenario where the bacteria population has approached the source molecules of interest. The outcome of the response and distribution of the entire population can be easily observed. The microfluidic device is used as a further accurate measure of the bacteria population’s ability to diffuse. Utilizing the design from Stocker et al [14, 15], we made the microfluidic device restrict the distribution of the bacteria population into a specific area. As the experiment begins, bacteria are allowed to swim freely from their original location. We can then calculate diffusion ability by measuring the resulting population distribution over time.

Using a microfluidic device, we tested the effect of “brake components” on population diffusion rate at different AHL concentrations. For each “brake component”, a characteristic equation is given to determine the relationship between diffusion rate and the activation level of such components. The activation level is relatively quantified by measuring the expression level of the green fluorescent protein (GFP) in front of the components. Through the characterization of the components, we anticipate that these components can be used in other promoter systems besides the quorum sensing system used in this study. Further, by simply measuring the promoter systems’ GFP expression level in advance, one can combine the promoter system with the appropriate components chosen in the library to design adequate genetic circuits suitable for searching particular molecules at a specific concentration.

## Materials and Methods

### Construction and verification of the basic circuit

In this study, synthetic genetic circuits are designed to reengineer *E*. *coli* to search for AHL. Here, several experiments were carried out to demonstrate that components used in the circuits worked successfully. The performances of the complete circuits were also tested to show that components work normally when integrated together in complex circuits.

**Fig 1:**
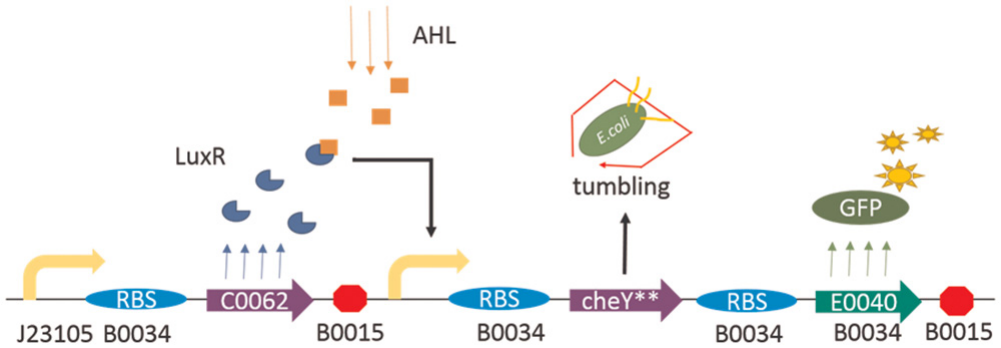
The basic design scheme of the synthetic circuit. Fig 1 shows the basic design scheme of the synthetic circuit used in this study. For figures throughout this study, arrows that turn right denote promoters, almonds denote RBS, arrows denote genes of functional proteins, and octagons denote terminators. If there is no additional explanation, genes used in this study are from the MIT Registry of Standard Biological Parts (database available online at http://parts.igem.org/Catalog). In the figure, LuxR protein, expressed constitutively by a weak promoter-RBS pair (J23105-B0034), is responsible for inducing expression of CheY** and GFP at pLux when AHL is combined. CheY** can cause the bacteria to tumble continuously while GFP exhibits green fluorescence, which aids measurement.

The basic circuit structure (named B1chey) is shown in Fig 1. The LuxR protein, expressed by a weak constitutive promoter (J23105), is responsible for inducing expression of GFP and CheY** at pLux when AHL is added. Expression of GFP enables us to quantify the protein expression levels induced by the pLux promoter. The cheY gene is amplified from *E*. *coli* MG1655 genomic DNA and modified at two positions by PCR technique (primers are shown in Table A in S1 File), which yielded the double mutant CheY**.

**Fig 2:**
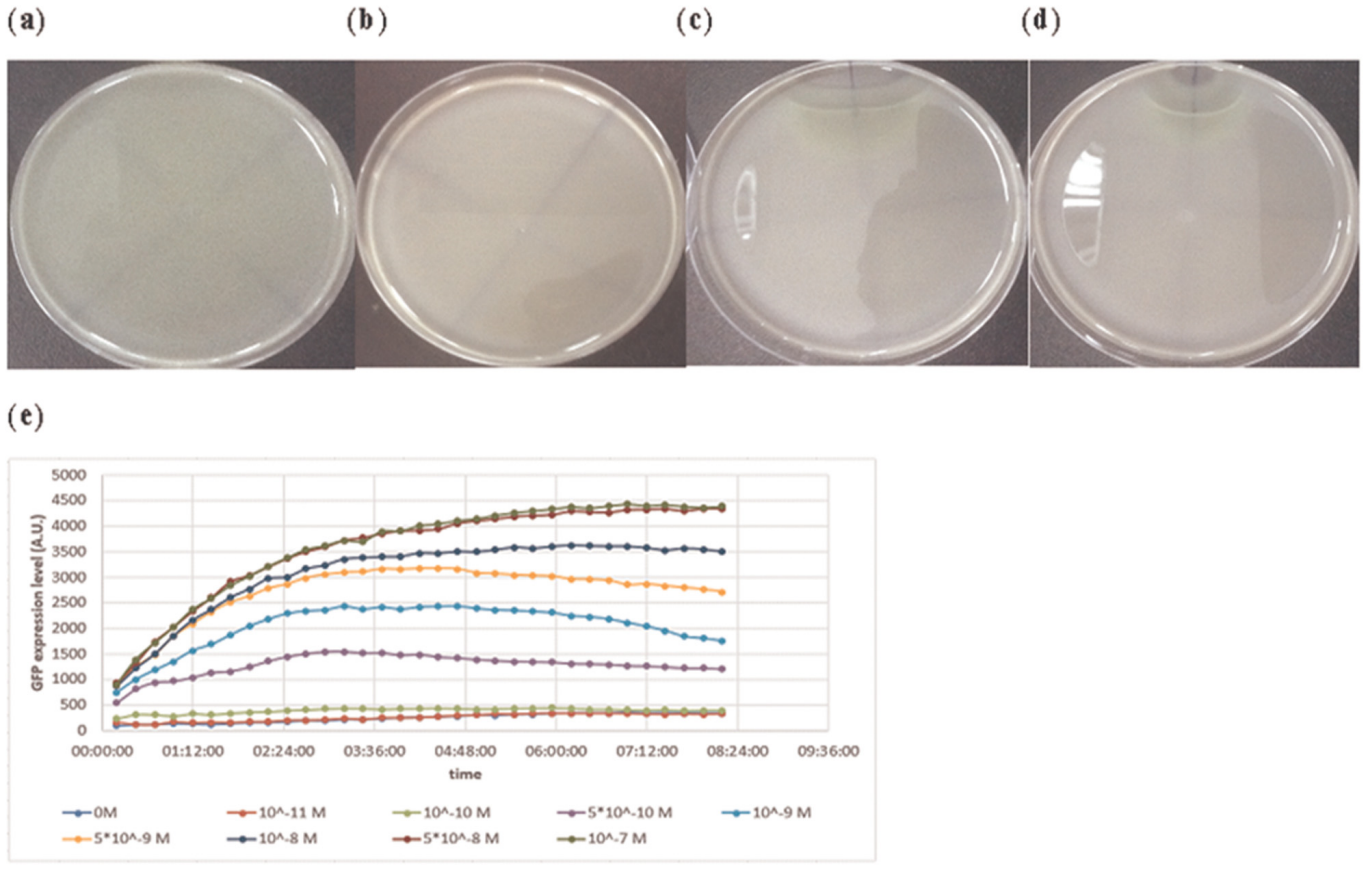
Verifying the performance of the synthetic circuit in Fig 1 in response to AHL. The searching ability induced by the circuit was tested by swarm assays. Without AHL, *E*. *coli* with the synthetic circuit in Fig 1 distributed evenly across the semi-solid LB agar plate in (b), as seen for *E*. *coli* with the control circuit in (a). While one microliter of 10^−3^ M and 10^−4^ M of AHL were added on the upper plate in (c) and (d), respectively, the *E*. *coli* stopped around the AHL sources and started producing GFP. The relationship between the efficiencies of the plux promoter and the concentration of AHL can be measured by the expression level of GFP. The result is given in (e).

In our design, the Chey** gene plays a central role in affecting *E*. *coli* mobility. We tested the performance of the Chey** gene using the swarm assay. *E*. *coli* MG1655 is transformed with circuit B1chey shown in Fig 2(a). The information of all *E*. *coli* strains used in this work is described in S1 File. Circuits with constitutive expression of GFP (J05I13504) were sent into *E*. *coli* MG1655 as a control. *E*. *coli* were cultivated in an LB medium and diluted to O.D. 0.05 before being dropped onto the semi-solid LB agar plate. After being cultivated for 16 hours, one microliter of 10^−3^ M, 10^−4^ M, or 0 M AHL was dropped beside the *E*. *coli* colonies. The colonies were then cultivated overnight. The result is shown in (Fig 2a–2d). *E*. *coli* with control circuits evenly diffused over the semi-solid LB agar plate. Without AHL, *E*. *coli* with circuit B1chey also distributed evenly. As AHL was added, the *E*. *coli* colonies diffused outward until they detected AHL and stopped around the AHL sources. The higher the concentration of the AHL sources, the wider the area where *E*. *coli* stopped moving forward. In line with our design, once *E*. *coli* stopped near the AHL sources, the *E*. *coli* population started accumulating and producing GFP. As a result, the genetic synthetic circuit is proven to make the *E*. *coli* population locate a specific area and execute the task they were engineered to perform.

For the same circuit, to quantify the expression level of the lux system in response to different AHL concentrations, an Elisa Reader measured GFP for 12 hours after adding AHL. The result is shown in Fig 2(e). After AHL was added, the expression of GFP gradually rose with time and achieved a steady state after 4 hours. With incremental AHL concentrations, GFP expression level also increased, as expected. The expression starts rising obviously as the AHL concentration exceeds 10^−10^ M and gradually saturates as the concentration reaches 5 × 10^−8^ M. Therefore, the most sensitive concentration range of the system to AHL is between 10^−10^ M and 5 × 10^−8^ M.

### Improvement of the circuits’ performance by constructing the “brake components” library

In the previous section, the distribution of *E*. *coli* populations in response to different AHL concentrations was demonstrated by observing diffusion on semi-solid agar plates. The result shows that *E*. *coli* stopped around the AHL sources and started expressing GFP as expected. However, *E*. *coli* could hardly invade the center of the sources. The area that constrained *E*. *coli* from entering further was affected by the concentration of the sources. The higher the concentration, the wider the area that develops. This phenomenon is due to the fact that the circuit responds intensively when the AHL concentrations rise above certain values and deprive the *E*. *coli* of almost their entire movement ability. Therefore, there is a specific threshold of AHL concentration above which *E*. *coli*’s production of TetR (tetracycline repressor) or CheY** will saturate. Once the *E*. *coli* population meets the threshold isopleth, the population stops and forms a border area shown in Fig 2. It is the hyperactivity of the circuits that makes our designs too sensitive to deal with high concentration cases.

To make our circuit less sensitive, one can choose to modify the CheY** structure. If CheY** were less active, *E*. *coli* might retain its basal movement ability and keep moving in the area with an AHL concentration above the threshold. However, to modify a protein properly is a difficult task since it is difficult to predict the effect of various mutations. Here, we chose the following synthetic biology method to solve this problem.

**Fig 3:**
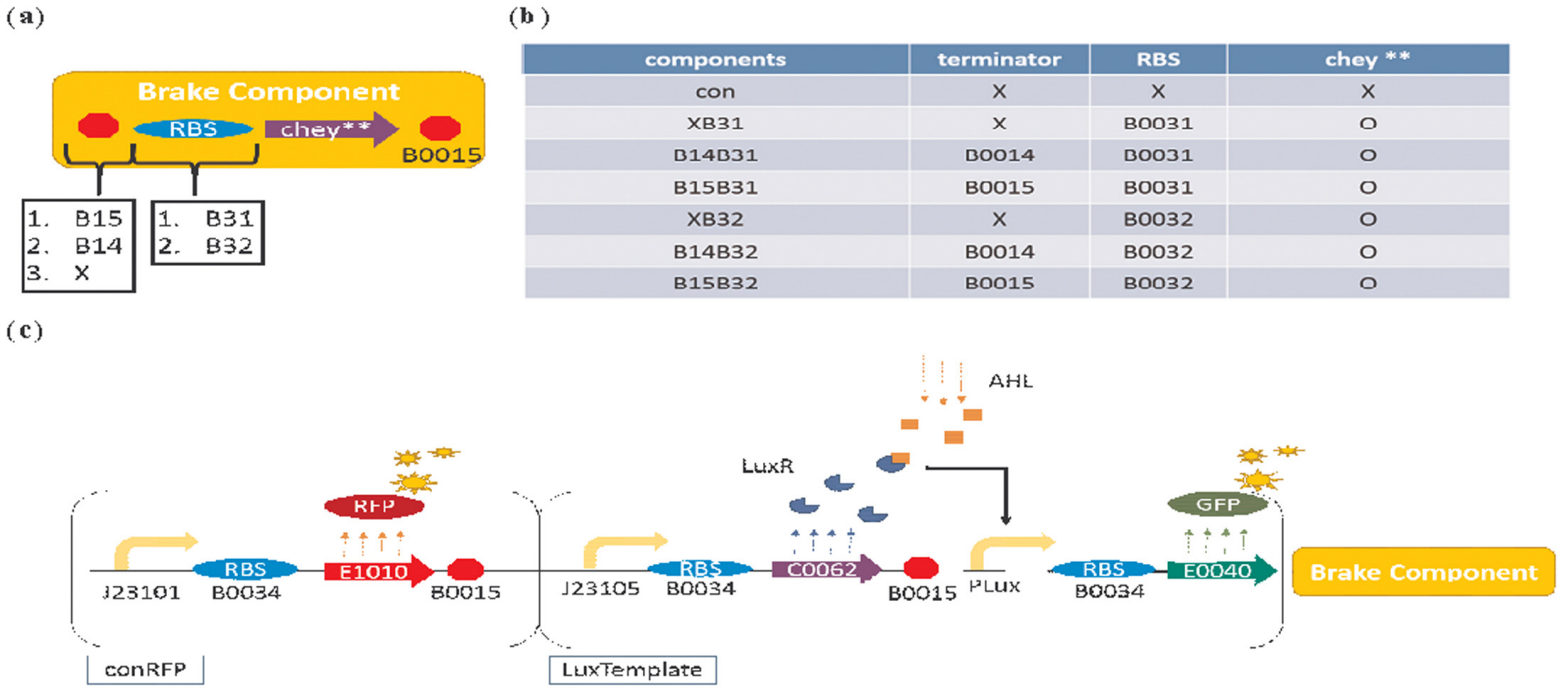
Improving the basic circuit by constructing a series of “brake components”. A basic “brake component” library in (b) was constructed to endow the standard circuit in (c) with various sensitivities to AHL. The structure of a “brake component” is shown in (a). A terminator and an RBS are used to regulate the expression level of the downstream CheY** protein by influencing the transcription and translation efficiency of CheY**, respectively. By choosing different terminators or RBSs, the “brake component” library in (b) can be constructed. The brake component with the part (terminator, RBS, or CheY**) is represented by O) while X represents without the part. Finally, the standard circuit in (c) is constructed by selecting specific brake components in response to different situations. This circuit is composed of three parts: (i) conRFP expresses RFP constitutively to facilitate the observation of the bacterial distribution; (ii) LuxTemplate detects AHL and induces downstream gene expression; and (iii) the aforementioned “brake component”.

First, circuits are modified by three ways to yield six combinations of components as shown in (Fig 3a and 3b). First, the Chey** gene is placed at the last position of the lux operon.

Second, the Chey**-related RBS is replaced by two weaker ones: B0031, whose efficiency when compared with B0034 is 0.07; and B0032, whose efficiency when compared with B0034 is 0.3. The data above were acquired from MIT Registry of Standard Biological Parts [16].

Third, three sequences with different terminating abilities are placed in front of the Chey**-related RBS: None; B0014 with 60.4% termination efficiency; and B0015 with 98.4% termination efficiency. The data above were acquired from MIT Registry of Standard Biological Part [17].

In addition, a “con” component, which lacks a terminator, RBS, and CheY** gene, was constructed as a control. The components were then combined with an RFP expressing circuits (conRFP) and a Lux-promoter-activated circuit (LuxTemplate) containing a GFP gene, which served as an index for the transcription and translation level. The complete circuits are shown in Fig 3(c), which is now ready for observing bacterial population distribution under a fluorescent microscope and measuring the GFP expression level by an Elisa Reader.

### Verification of the function of the components by swarm assays

In this section, we simply tested the modified circuits in Fig 3(c) by the swarm assays. Three of the components, XB32, B14B32, and B15B32, which contain different terminators, were chosen to demonstrate the circuits’ performance. We anticipate that different amounts of CheY** gene can be transcribed according to the termination efficiency of the terminators. A strong terminator, B0015, can block most of the transcription, which restricts the amount of the CheY** protein, unless considerable AHL is detected. Thus, component B15B32 is expected to have the weakest sensitivity. Conversely, the components B14B32 or XB32, with weak or no terminator, show high sensitivity.

**Fig 4:**
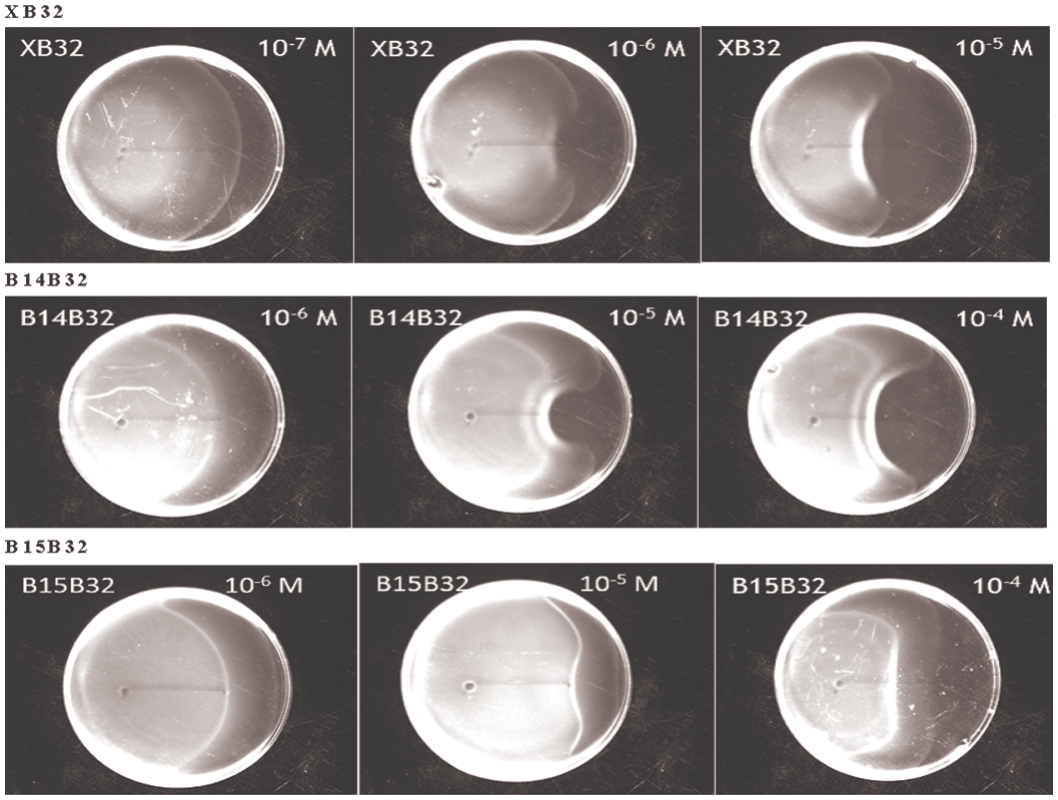
Verification of the brake components by swarm assays. Swarm assays were performed to verify the function of different brake components. Standard circuits containing three brake components, XB32, B14B32, and B15B32, were sent into *E*. *coli* strain RP437. After being cultivated in M9 medium, *E*. *coli* were dropped at the left end of the line on the semi-solid agar made with M9 medium. Simultaneously, a 1.5 microliter with a specific concentration of AHL was dropped at the right end of the line on the agar (4 cm away from the *E*. *coli*). The distribution of the *E*. *coli* population was observed after 20 hours of cultivation.

We verified the aforementioned components by swarm assays using *E*. *coli* strain RP437, which is widely used in chemotaxis and swimming studies. *E*. *coli* and AHL were dropped on the agar spanning a fixed distance. After sufficient time for diffusing, the results are shown in Fig 4. In the figure, we can see that each component has its ideal concentration range for molecule detecting. Taking XB32 for example, the bacteria population can appropriately surround a drop of 10^−6^ M AHL. However, in the case that the concentration is lowered to 10^−7^ M, the bacteria can hardly detect the source of the AHL. In another case, while the concentration is increased to 10^−5^ M, the bacteria population intends to stop too far away from the molecule source. Similarly, both the components B14B32 and B15B32 have the same property. The appropriate concentration for molecule detection is 10^−5^ M for B14B32, whereas the appropriate concentration for molecule detection is between 10^−4^ M and 10^−5^ M for B15B32. This corresponds to our expectation that sensitivity increases with decreasing termination efficiency. As a result, we confirm that our design is effective to engineer bacteria to search for different concentrations of AHL.

## Results

### Measurement and identification of the “brake components”

In the previous section, we verified the circuit design method in this study and improved it by several modifications. Further, we have proven that the modifications are effective by swarm assays. However, it is worth noting that the swarm assays can only verify the circuits’ property qualitatively. The bacteria population was placed on semi-solid agar and allowed to search for the AHL source, which was organized by dropping a particular amount of AHL on the agar. The spatiotemporal distribution of the AHL is undetectable and unpredictable. Hence, although we know the initial amount of the source and can observe the distance between the center of the source and the place where the population stopped, we cannot tell the exact concentration which stops the population from diffusing. Therefore, the microfluidic technique is applied in this study to measure diffusion rate of the bacterial population under different AHL concentrations.

Here, the experimental methods to measure the diffusion rate include the mathematical principle, fabrication of the microfluidic device, experimental procedure, and data processing. Following these methods for the circuit in Fig 3(c), we observed the relationship between AHL concentrations and the corresponding population diffusion rate, which generated the characteristics of each “brake component”. To compare components more objectively, we adopted a relative quantification method. The expression levels of the components were represented by the expression levels of the upstream GFP. A sigmoid-like function was thus constructed to simulate the relationship between GFP expression and population diffusion rate. This function can provide useful information, such as effective region and saturation point of each component, which we can use to compare components in the library and select appropriate circuit design. Further, different mutant levels of CheY protein were also tested. We anticipate that the mutant levels may make small adjustments to the components and enrich our components library.

### Methods for measuring bacteria population diffusion rate

In this section, we describe the method for measuring diffusion rate of the bacterial population by a microfluidic device. There are several advantages to measuring diffusion rate using a microfluidic technique: (i) channels with micrometer-scale are fabricated rapidly and precisely; (ii) more accurate flow can be produced; and (iii) devices can be made from PDMS, which is transparent, such that cells can be directly observed by a microscope [14, 18]. By utilizing the design from Stocker et al., we constructed a microfluidic device capable of locating bacterial populations in a particular area. By observing bacterial distribution spatiotemporally, we calculated the diffusion rate of the population. In the following paragraphs, the aforementioned sections containing the mathematical principles, fabrication of the microfluidic device, experimental procedure, and data processing will be introduced in order.

So far, a number of mathematical models describing bacteria movement have been established. At the population scale, the most widely used model is that of Keller and Segel [19], which provides an expression for the flux (J) of bacteria:

$$\text{J}=-\mu \frac{\partial \text{B}}{\partial \text{x}}+V_{C}B$$ (1)

where B(x,t) denotes the concentration of bacteria, μ is the random motility coefficient that indicates the diffusivity of the population owing to their random walk behavior, and *V*_*C*_ is the chemotaxis velocity resulting from their preferred movement toward attractants or away from repellents. Substituting the above equation into the total cell number density conservation equation $\frac{\partial \text{B}}{\partial \text{t}}=-\frac{\partial J}{\partial x}$ [20] yields the bacterial transport equation:

$$\frac{\partial \text{B}}{\partial \text{t}}=\frac{\partial }{\partial x}\left(\mu \frac{\partial B}{\partial x}\right)-\frac{\partial }{\partial x}\left(V_{C}B\right)$$ (2)

In this study, we characterized components using *E*. *coli* strain RP5232, which lacks the CheY gene. This strain was chosen for two reasons. First, the phosphorylated CheY protein can influence the rotating direction of the flagella and make *E*. *coli* tumble. Without CheY, *E*. *coli* that swim smoothly can be easily observed to switch between their original and tumbling state induced by the “brake components”. Second, in the absence of the CheY gene, the chemotactic pathway is blocked so that no chemotactic behavior will affect the measurement of the diffusion rate. Therefore, since *V*_*C*_ = 0, Eq (2) will reduce to the diffusion equation:

$$\frac{\partial \text{B}}{\partial \text{t}}=\frac{\partial }{\partial x}\left(\mu \frac{\partial B}{\partial x}\right)$$ (3)

Solving the partial differential equation yields:

$$\text{B}\left(\text{x},\text{ t}\right)=\frac{e^{\left(-\frac{x^{2}}{4\mu \left(t+t_{0}\right)}\right)}}{\sqrt{4\mu \left(t+t_{0}\right)}}$$ (4)

Based on Eq (4), we established a one-dimensional band of *E*. *coli* using the microfluidic device. By measuring the concentration (B) of *E*. *coli* at different positions (x), we compute *μ*(*t* + *t*_0_) by fitting the measurement to Eq (4). Calculating *μ*(*t* + *t*_0_) at different time points (t), we can obtain the diffusion rate *μ* by linear regression.

**Fig 5:**
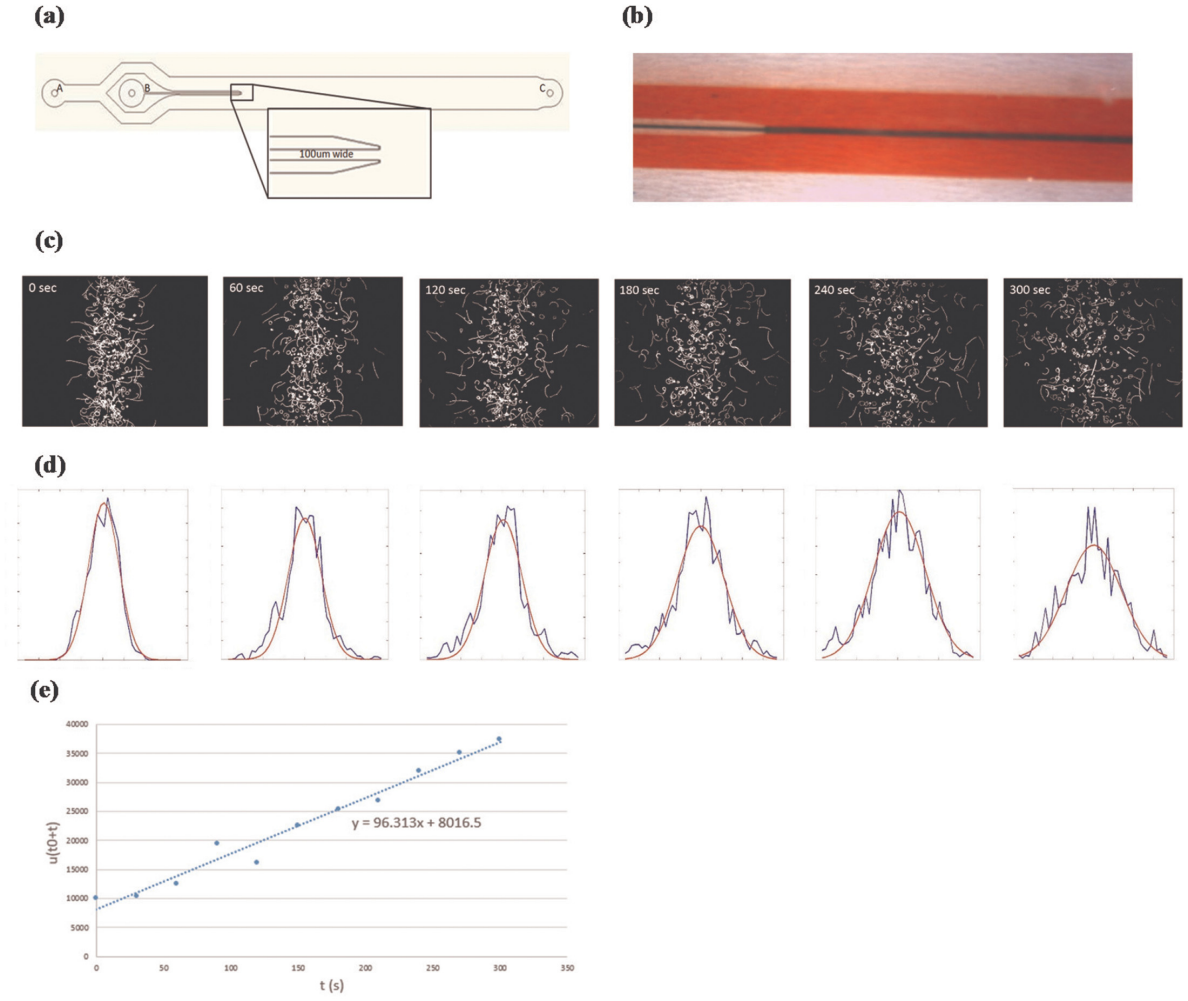
The methodology for measuring bacterial population diffusion rates. Figure (a) shows the pattern of the microfluidic device. In the figure, A and B are the input of the medium and bacteria, respectively, and C is the output of the microfluidic device. The outer flow will restrict the distribution of the inner flow as shown in (b) (displayed by red and blue coloring). Once both flows stop, the *E*. *coli* in the inner flow starts to diffuse outward. The trajectory of the *E*. *coli* at different time points can be recorded by microscope in (c). The distribution of the bacteria population over time can also be calculated in (d). The bacterial population diffusion rate is obtained by handling the standard deviation of the population distribution over time by linear regression, as shown in (e). In this case, the diffusion rate is 96.313 *μm*^2^/*s*.

Next, to demonstrate the experimental procedure, we measure *E*. *coli* strain RP5232 containing circuit “conRFP-LuxTemplate-con” without AHL being added as an example. First, the microfluidic device in Fig 5(a) was constructed. A and B in Fig 5(a) are the inlets for medium and bacteria, respectively. The flows of bacteria and medium converge at the end of the micro-injector and outflow from outlet C. As liquids were pushed into inlets A and B under a stable pressure, liquid from inlet B was sandwiched in the middle of the channel shown in Fig 5(b) (displayed by red and blue coloring). When the pressure was turned off, both the flow stopped and the band of *E*. *coli* population started to expand to both sides. To record the distribution of the population, red fluorescence protein produced by *E*. *coli* was captured by a fluorescence microscope. We took bursts of 25 pictures over 5 seconds interspaced with 30 second intervals without imaging.

Finally, coupling these 25 pictures gives the trajectory of *E*. *coli* over 5 sec, as shown in Fig 5(c). Distribution (B) of *E*. *coli* at different positions (x) was computed by summing up the trajectory of pixels, which was x micrometers from the center axis and normalized by the total number of pixels of the trajectory. Distribution (B) was then fitted by a genetic algorithm (GA) to Eq (4), which yielded values of *μ*(*t* + *t*_0_). The fitting results are shown in Fig 5(d). Blue and red lines denote the experimental results and their fitting results, respectively. Diffusion rate *μ* was acquired by handling values of *μ*(*t* + *t*_0_) over time by linear regression shown in Fig 5(e). In this case, *μ* = 96.3 *μm*^2^/*s*.

### Measurement of the “brake components”

**Fig 6:**
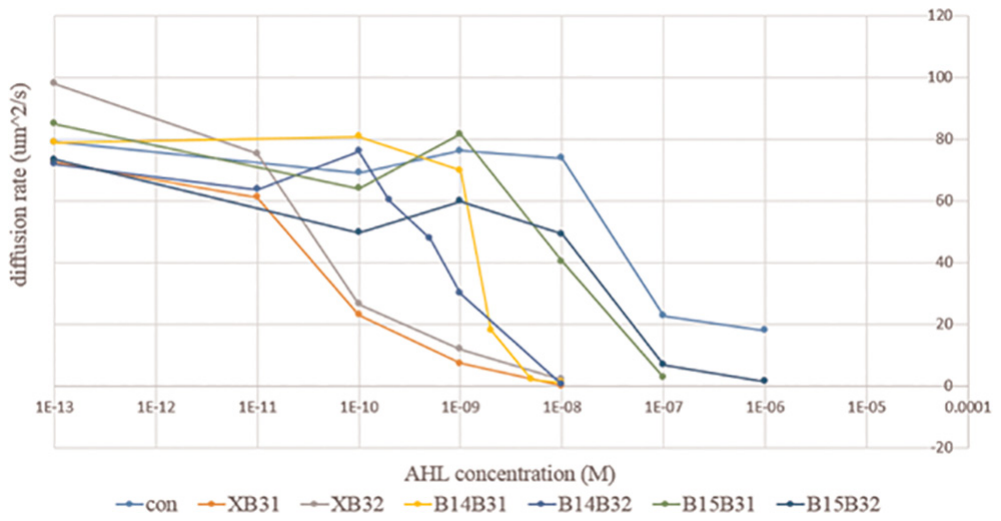
Comparing the effect of each brake component in response to different AHL concentrations. All the brake components in the component library in Fig 3(b) were combined with a standard circuit and sent into *E*. *coli* strain RP5232 to measure their effect on population diffusion rate by the microfluidic device. Each line represents how a specific component influences the diffusion of bacteria in response to different AHL concentrations.

Based on the methods, the effect of circuits with different “brake components” shown in Fig 3(a) on bacterial population diffusion rates were measured. For each component, several AHL concentrations were added to test the performance under different conditions and components’ sensitivities. Each result is shown in Fig A in S1 File and summarized in Fig 6.

In Fig 6, the “con” circuit, whose “brake component” is blank, is the control of the experiment. Notably, without the production of the CheY** protein, the diffusion rate drops to the basal level at high concentrations of AHL (exceeding 10^−7^ M). This suggests that the mass expression of GFP may reduce bacteria mobility. The six components in the library show a similar tendency toward stopping the *E*. *coli* completely as AHL concentration increases. As expected, the six components succeed in displaying different sensitivities toward AHL. By comparing their performance, we conclude that the sensitivities of the components mainly depend on their terminator in front of the RBS and Chey**. Components without a terminator (XB31, XB32) present the highest sensitivity, components with a weak terminator (B14B31, B14B32) present normal sensitivity, and components with a strong terminator (B15B31, B15B32) present the lowest sensitivity.

RBSs also provide minor modifications to the components. However, according to this figure, strength does not differ between the two RBSs. In cases without a terminator (XB31, XB32) and with a strong terminator (B15B31, B15B32), components containing B32 present lower sensitivities. While in another cases (B14B31, B14B32), components containing B32 present higher sensitivities. The difficulty in distinguishing the effect derived from different RBSs can be explained by inconsistencies between component activation levels. In this study, GFP was put in front of the “brake components” to quantify expression between components. Thus the GFP expression levels denote the components’ activation level. Fig C in S1 File shows the GFP expression levels of the control circuits during the diffusion experiment. The concentration region of AHL to which the circuits are most sensitive is between 10^−10^ M and 10^−8^ M. That is, in this region, any fluctuation to the promoter system tends to be significantly amplified. To demonstrate this phenomenon, we show in Fig C in S1 File the GFP expression level of all components during the experiment. On the condition that no AHL was added or that AHL reached saturation (10^−8^ M), variation between components was negligible. However, GFP expression levels varied obviously when AHL concentrations (10^−10^, 10^−9^) were near the most sensitive region. For both cases, the highest value is three times more than the lowest one. The reasons for the variation might be due to intrinsic factors, such as bacterial individual differences or different secondary structures of mRNA derived from different components that may influence translation efficiency, or extrinsic factors like pipetting errors when preparing extremely low concentrations of AHL.

Briefly speaking, while comparing performance among different components, the activation levels of components tend to be inconsistent, especially when AHL concentrations are near the most sensitive region. Moreover, this region (10^−10^ M ~ 10^−8^ M) covers the range where many components perform their function significantly, which makes comparison difficult. To solve this problem, we compare and define each component directly through their activation levels, instead of AHL concentrations. The method and results will be discussed in the next section.

### Constructing a mathematical model to identify the “brake components”

In this section, we establish a mathematical model for describing the relationship between component activation levels and their corresponding bacterial population diffusion rates. By characterizing by activation levels, we can directly compare components by the well-measured GFP expression levels, as well as utilize components in other promoter systems without re-characterization. Through measuring the promoters’ GFP expression levels in advance, we can estimate the consequent performance of the circuits when selecting different components. We expect that this may contribute to circuit design substantially.

The experimental data suggested that, for every component, the curves of diffusion rates remained high and smooth when the activation levels were below specific values, above which the rates decreased abruptly. The main differences among components were when and how the diffusion rates fell. To describe this desired diffusion rate phenomenon, we set up a sigmoid-like mathematical model:

$$\mu \left(\text{CAL}\right)=\frac{\mu_{0}}{1+\text{exp}\left(a\left(CAL-b\right)\right)}$$ (5)

where μ and *μ*_0_ denote diffusion rate and nominal diffusion rate when no AHL was added, respectively, CAL is the abbreviation of components’ activation levels, which are represented by the GFP expression levels, *a* indicates the decreasing degree of the curve, while *b* indicates the activation levels required for the diffusion rate to fall to half of its initial value *μ*_0_. Both *a* and *b* can be calculated by fitting the experimental data to the model by genetic algorithm.

**Fig 7:**
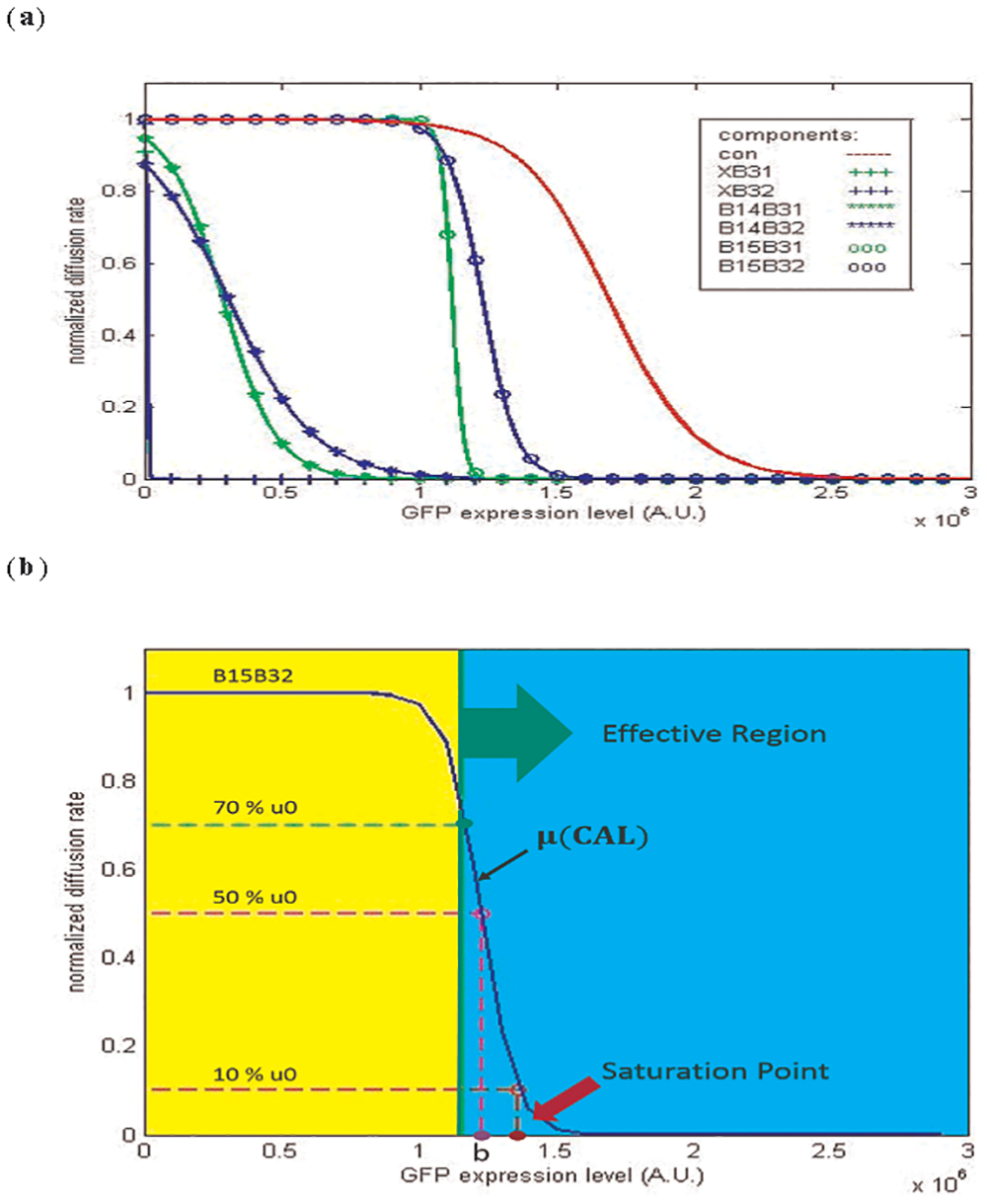
Representation of components characterized by Eq (5). The results of fitting the experimental data of each brake component to Eq (5) are shown in Fig B in S1 File and summarized in (a). The x-axis of the figure represents the GFP expression level of the circuit, which can be viewed as the components’ activation levels (CAL) in Eq (5). Based on Eq (5), some criteria of the components can be defined to facilitate the comparison among components. In (b), the “Effective Region” indicates a range of CAL upon which the diffusion rate of the population is less than 70% of the initial diffusion rate. The “Saturation Point” denotes the CAL that makes the diffusion rate of the population equal to 10% of the initial diffusion rate.

Fitting the experimental data of each component to the model yielded the results shown in Fig B in S1 File. To facilitate comparison, we normalized μ(CAL) by *μ*_0_ and summarized this in Fig 7(a). First, in Fig 7(a), the curve of the control circuits drops significantly after the GFP expression level exceeds a value of approximately 1.5 × 10^6^ (A.U.), beneath which the curves of all components have already decreased to their basal levels. This indicates that the decreased diffusion rate is mainly due to component expression rather than mass expression of GFP protein. Further, similar to Fig 6, the six components can be obviously divided into three categories according to their terminators. As GFP expression levels rise, components without a terminator (XB31, XB32) cause diffusion rates to drop first, followed by components with a weak (B14B31, B14B32) and then strong (B15B31, B15B32) terminator. This is consistent with results in the previous section. Moreover, in the previous section, the difference between the two RBSs was not obvious. Here, by comparing the fitted value “b” in Fig 7(b), we find in the cases with fixed terminators, components with RBS B31 always have a smaller “b”, which indicates that lower component activation levels are required to meet half their diffusion rates. Thus, components with B31 are slightly more sensitive than those with B32.

Characterizing components provides some useful information for circuits design. To facilitate the selection of the proper components, we define some criteria, shown in Fig 7(b), which contribute to comparison among components. “Effective region” denotes the ranges of activation levels in which the diffusion rates are less than 70% of *μ*_0_. This suggests that, for a component, the diffusion rate of bacteria will not be affected until the component’s activation level enters its effective region. “Saturation point” denotes the values of activation levels upon which the diffusion rates drop to one-tenth of their initial diffusion rates *μ*_0_. Once the component’s activation level exceeds the saturation point, the increment will make no difference to diffusion rate.

These criteria provide some considerations for circuit design. For example, it is worth noticing that when dealing with a promoter that has a tendency to leak, components whose effective regions cover the leakage level of such promoter systems are not suitable for this situation. Otherwise, the components will be activated consistently, irrespective of whether or not the promoter is induced. In another case, when a component is chosen in order to make the bacteria trace a specific concentration of molecule, the most appropriate option among the components library is the one whose saturation point is slightly lower than the expression level of the promoter under such concentrations. Thus, the bacteria will express adequate levels of the brake component and stop as they move into the area containing the expected concentration.

Finally, we list the information of each component, including the fitted value (*a*, *b*) and the above-mentioned criteria in Table 1. In addition, the application of the table and the mathematical model to circuits design will be discussed in future research.

**Table 1:** Information of the identified “brake components”.

Components	a	b	Effective region	Saturation point
con	6.38×10^−6^	1.69×10^6^	CAL>1.55×10^6^	2.03×10^6^
XB31	2.67×10^−4^	1.01×10^4^	CAL>6.92×10^3^	1.83×10^4^
XB32	4.12×10^−4^	1.06×10^4^	CAL>8.58×10^3^	1.60×10^4^
B14B31	1.02×10^−5^	2.84×10^5^	CAL>2.01×10^5^	5.00×10^5^
B14B32	6.37×10^−6^	3.06×10^5^	CAL>1.73×10^5^	6.51×10^5^
B15B31	4.75×10^−5^	1.12×10^6^	CAL>1.10×10^6^	1.16×10^6^
B15B32	1.62×10^−5^	1.23×10^6^	CAL>1.17×10^6^	1.36×10^6^
XB32CheY	1.50×10^−4^	4.12×10^4^	CAL>3.48 ×10^4^	5.51 ×10^4^
B14B32CheY	5.00×10^−4^	1.06×10^6^	CAL>1.05 ×10^6^	1.11×10^6^
XB32CheYD13K	4.42×10^−4^	7.04×10^3^	CAL>5.13×10^3^	1.20×10^4^
B14B32CheYD13K	1.22×10^−5^	2.16×10^5^	CAL>1.46×10^5^	3.96×10^5^

### Enrichment of the “brake components” library by mutations

CheY^D13K Y106W^ (CheY**) was chosen in this study to induce tumbling in *E*. *coli*. In the absence of CheA, a kinase to phosphorylate CheY, CheY** is still active without phosphorylation. Even when CheZ, a protein known to accelerate the dephosphorylation of CheY-P, was expressed in high levels, Chez failed to respond to CheY** [13]. Studies showed that when CheY** binds FliM_16_, a peptide derived from the flagellar motor switch FliM, CheY** has a conformation similar to $\text{Be}F_{3}^{-}$ -activated wild type CheY, which is a stable analog of the phosphorylation-induced conformation. In the absence of FliM, CheY** has a conformation similar to unphosphorylated wild type CheY. In short, CheY** is suitable to convert between its active and inactive conformation in the absence of phosphorylation or dephosphorylation [12].

Recent studies have reported that there are other mutations that show individual effects. For example, similar to CheY**, CheY^*D*13*K*^ is active in the absence of phosphorylation. CheY^*Y*106*W*^ and CheY^*I*95*V*^ are hyperactive when they are phosphorylated [21, 22]. Compared with the previous two mutations, CheY^*Y*106*F*^ has lower activity, which causes a decreased tumble signaling, whereas CheY^*Y*106*L*^, CheY^*Y*106*I*^, CheY^*Y*106*V*^, CheY^*Y*106*G*^ and CheY^*Y*106*C*^ show no activity and cause smooth swarm [22]. Introducing the various mutations of CheY, the “brake components” library can be efficiently enriched by simply carrying out the mutations based on the existing circuits.

Here, we expanded two more versions of components XB32 by adopting different mutant levels. Utilizing wild type CheY and CheY^*D*13*K*^ we could create two new components named XB32CheY and XB32CheYD13K, respectively. We tested the characteristics of these components by previous methods. The results are shown in Table 1. The wild type version of XB32 has a larger activation level “b” than the others. This suggests, as expected, that it makes the component less sensitive. Surprisingly, XB32CheYD13K shows the smallest activation level “b”, which indicates that the CheY^*D*13*K*^ version is even more sensitive than the double mutant version, CheY**. Therefore, all the components in the library can be altered to a more sensitive (CheY^*D*13*K*^) or less sensitive (wild type) version through this method.

### Simulation of the proposed genetic circuit

The purpose of this section is to simulate the operation of the genetic circuits. Two kinds of model will be discussed in this section. First, we will construct the model that could establish the relationship between AHL concentration and the corresponding protein expression level to show component activation levels (CAL) of the “brake components”. Coupling this model with identifying components in Eq (5), we are able to simulate the performance of the complete circuits, i.e., to simulate the bacterial population diffusion rate at all AHL concentrations. This method is not restricted to the lux promoter system in this study. When the components are transplanted to other promoter systems, we can simulate the complete circuits’ performance at all concentrations of the new stimulus by identifying the new promoter systems. Combining the identification of the components with the model, we can simulate the diffusion rate at all AHL concentrations. The simulated results are then compared with the experimental ones.

However, for each component, how the parts (terminators, RBSs and different CheY mutations) influence the parameters in Eq (5) (i.e. “a” and “b”) has not yet been discussed. The second model is established to describe the relationship between the components activation levels (CAL) and the bacterial population diffusion rate. By highlighting the relationship between the factors and parameters, differences in parameters can be explained between components. More importantly, the characteristic of those uncharacterized components can be anticipated. That is, we are able to construct new components based on existing parts and simulate their properties without measuring each of them. For example, we introduced the wild type CheY and CheY^*D*13*K*^. The number of the components in the library can be tripled by replacing the CheY** in the existing components with the two new ones. The characteristics of the new components in the library can be anticipated by simulation. Combined with the model, enrichment of the library is beneficial for selecting the ideal components for detecting specific AHL concentrations when designing the circuits.

### Dynamic model of the synthetic circuit

In this section, we aim to establish the relationship between AHL concentration and the corresponding protein production generated by the lux promoter system. Considering the two factors that may affect transcription level, the amounts of transcription factor luxR protein and luxR-AHL complex, we construct the following dynamic equations to describe the performance of the genetic circuits in Fig 1(c) [23, 24, 25, 26]:

$$\left\{ \begin{array}{c} \;{\dot{x}}_{luxR}\left(t\right)=P_{J05B34}-\left(d_{luxR}+D\right)\cdot x_{luxR}\left(t\right)\\ \;{\dot{x}}_{GFP}\left(t\right)=P_{luxB34}\left(I,x_{luxR}\right)-\left(d_{GFP}+D\right)\cdot x_{GFP}\left(t\right)\\ \;P_{luxB34}\left(I,x_{luxR}\right)=P_{m,lux}+\frac{P_{M,lux}-P_{m,lux}}{1+{\left(\frac{K_{luxR}}{x_{luxR}^{*}\left(x_{luxR},I\right)}\right)}^{n_{lux}}}\\ x_{luxR}^{*}\left(x_{luxR},I\right)=\frac{x_{luxR}}{1+\frac{K_{AHL}}{I}} \end{array}\right.$$ (6)

where *x*_*luxR*_(*t*) and *x*_*GFP*_(*t*) denote the amount of luxR and GFP respectively, which are affected by the following factors: *P*_*J*05*B*34_ and *P*_*luxB*34_ denote the transcription and translation strength of the indexed promoter-RBS pair; and *P*_*J*05*B*34_ is a constitutive value, whereas *P*_*luxB*34_ varies according to the amount of *x*_*luxR*_ and *I*, which is the concentration of inducer AHL. The strength of the promoter-RBS pairs (*P*_*J*05*B*34_, *P*_*luxB*34_), as well as the degradation (*d*_*luxR*_, *d*_*GFP*_) and dilution rate (D), owing to cell division, control the amount of protein per cell. Further, the strength of *P*_*luxB*34_ can be described by the third equation in Eq (6). *P*_*m*,*lux*_ and *P*_*M*,*lux*_ denote the basal and maximum expression level of the promoter-RBS pair. *K*_*luxR*_ and *n*_*lux*_ are the binding affinity and binding co-operativity between the promoter and the luxR-AHL complex $x_{luxR}^{*}$, which is influenced by the amount of luxR, AHL, and the dissociation rate *K*_*AHL*_ between luxR and AHL.

During the experiment, if a sufficient time is provided for bacteria to respond to AHL (about four hours), the expression level of the protein will reach steady state in which the production rate of the protein is equal to its degradation and dilution rate. Thus, the above-mentioned dynamic model can be simplified to its steady state form as follows:

$$\{\begin{array}{c} \;x_{luxR}=\frac{P_{J05B34}}{\left(d_{luxR}+D\right)}\\ \;x_{GFP}\left(I\right)=\frac{P_{luxB34}\left(I,x_{luxR}\right)}{\left(d_{GFP}+D\right)} \end{array}$$ (7)

With the steady state equations, through measuring the amount of the proteins and referring to previous studies for some parameters (*d*_*luxR*_, *d*_*GFP*_, *D*), we can identify the remaining parameters (*P*_*J*05*B*34_, *P*_*m*,*lux*_, *P*_*M*,*lux*_, *K*_*luxR*_, *n*_*lux*_, *K*_*AHL*_) by a genetic algorithm and accomplish the dynamic model. The results of the identification and simulation are shown in the following section.

### Identification of the diffusion rate of bacterial population at all AHL concentrations

To identify the unknown parameters (*P*_*J*05*B*34_, *P*_*m*,*lux*_, *P*_*M*,*lux*_, *K*_*luxR*_, *n*_*lux*_, *K*_*AHL*_) in Eq (7), two experiments were performed to acquire the protein expression level derived from the two promoter-RBS pairs, J23105B0034 and PluxB0034.

**Fig 8:**
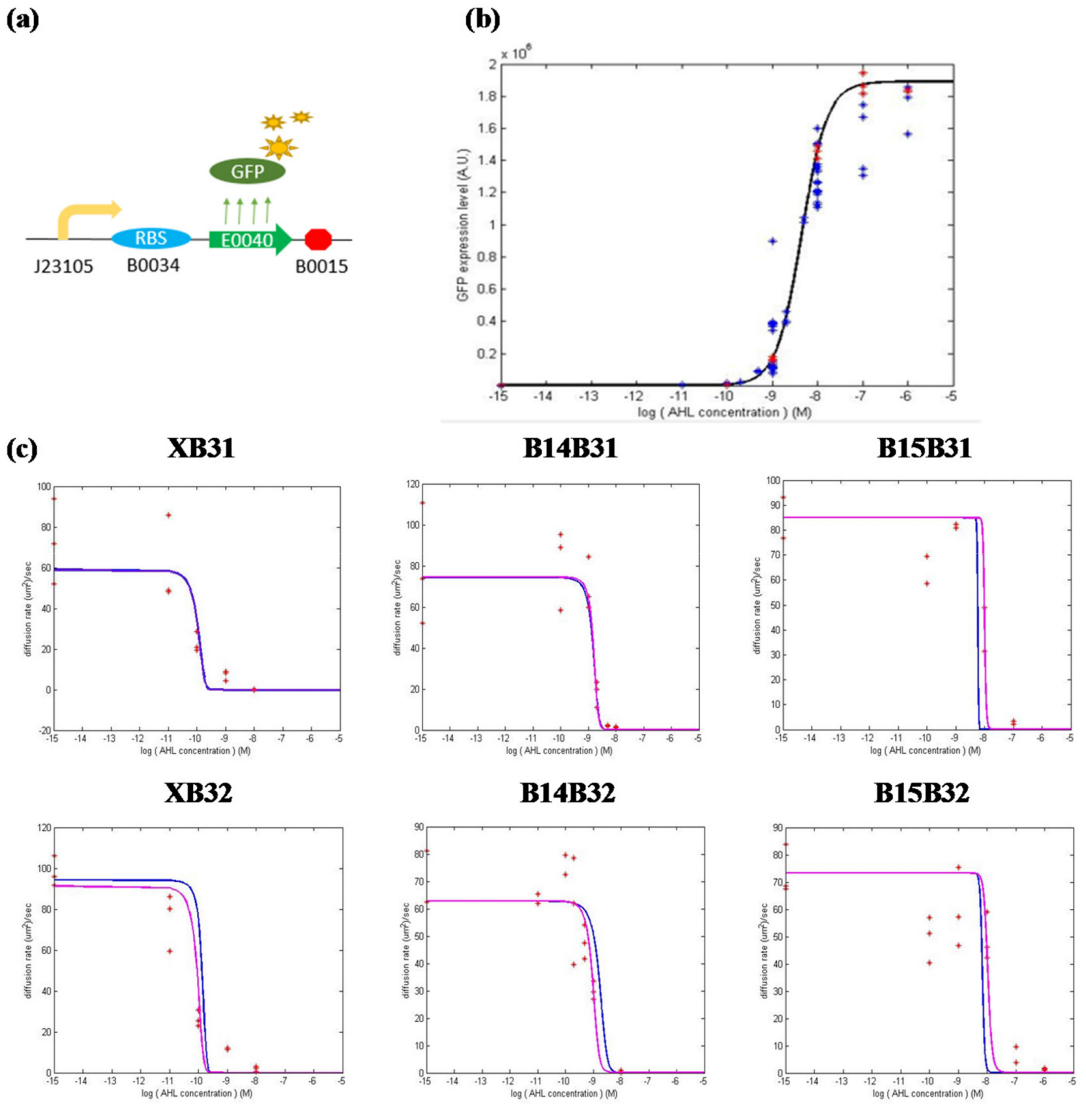
Simulating “stimulus vs. CAL” and “stimulus vs. diffusion rate” based on the dynamic model. In (a), the circuit was used to identify the strength of the constitutive promoter-RBS pair J23105B0034. The experimental data of the standard circuit with the “con” component was used to identify the parameters in Eq (7). In (b), the simulated equation, *x*_*GFP*_(*I*) in Eq (7), is represented by a black line, whereas the experimental result of the representative “con” component is represented by red stars. For the rest of the brake components in Fig 3(c), their experimental data are collectively represented by blue stars. In (c), the simulated (using the unified parameters identified from the “con” component) and experimental results for each brake component are represented by a blue line and red star, respectively. Based on the experimental results, the simulation can be improved (purple lines) by identifying the respective parameters for each component.

First, to identify the strength of the constitutive promoter-RBS pair J23105B0034, synthetic circuits shown in Fig 8(a) were constructed and sent into *E*. *coli* strain RP5232. To facilitate the measurement of protein production, the luxR protein was replaced by a GFP protein, which can be simply measured by an Elisa Reader. Under this condition, the degradation rate *d*_*luxR*_ in the first equation of Eq (7) must be altered, which yields $\;x_{luxR}=\frac{P_{J05B34}}{\left(d_{GFP}+D\right)}$. As a result, simply measuring the GFP expression level at steady state gives the strength *P*_*J*05*B*34_ of the promoter-RBS pair.

Second, to identify the parameters related to the AHL-activated promoter-RBS pair PluxB0034, the synthetic circuit in Fig 3(c) with the “con” component was selected to represent all circuits in the figure. We assume that the components behind the GFP sequence will make no difference to the expression level of the GFP protein. Hence, for all the circuits in the figure, the equation and its parameters describing the PluxB0034 system can be unified. To identify the parameters, *E*. *coli* strain RP5232 was transformed by circuits with the “con” components. Different AHL concentrations (*I*_*AHL*_) were added to the bacterial culture and the corresponding GFP expression levels (*x*_*GFP*_) at the steady state were measured. Further, *x*_*luxR*_ was calculated according to the first equation in Eq (7) whose *P*_*J*05*B*34_ was obtained from the first experiment. Based on the above information, the remaining parameters (*P*_*m*,*lux*_, *P*_*M*,*lux*_, *K*_*luxR*_, *n*_*lux*_, *K*_*AHL*_) can be estimated according to Eq (7).

To verify whether the model and the identified parameters corresponded to the experimental results, we next compare the simulated and experimental results, shown in Fig 8(b). The figure shows that the simulation accurately captures GFP expression in response to different AHL concentrations. In addition, we show the experimental results of all the components in Fig 3(c) in blue stars, which show that the simulation also succeeds in representing them.

Finally, we combine the simulation with the characteristics of the components, which links the amount of AHL and the consequent diffusion ability. The simulations in Fig 8(c) have unified these parameters to approximate the performance of each circuit and can be improved by identifying their own parameters, shown by purple lines. The improvement contributes to reducing the intrinsic factors that cause differences in GFP expression levels between circuits. That is, through identifying the promoter-RBS system, we can estimate the performance of the synthetic circuits at the design stage. Simulations can be further improved after constructing synthetic circuits.

### Constructing a mathematical model to simulate the characteristic of the “brake components” based on components’ composition

In the previous section, we constructed a mathematical model to describe the relationship between AHL concentration and protein production, which is expressed by the lux promoter system. Here, we further aim to establish the relationship between the above mentioned protein production, which also indicates the component’s activation levels (CAL), and the bacterial population diffusion rate.

The sigmoid-like equation in Eq (5) can appropriately depict the behavior of the bacterial population diffusion rate in response to CAL. Therefore, we established the model based on this existing equation. In the case of the component XB32 without a terminator in front of RBS and CheY**, Eq (5) can be viewed as the direct relationship between the relative amount of the CheY** protein and the bacterial population diffusion rate. Compared with XB32, other components can alter the transcription and translation efficiency of the CheY** protein, while the relationship between the amount of CheY** and bacterial diffusion rate remains the same. For example, combining terminator B0014 in front of XB32 makes B14B32. Therefore, the terminator can block a certain ratio of CheY** production. Thus, Eq (5) for B14B32 can be directly represented by the modified XB32’s equation whose CAL is divided by a specific value, which indicates the termination efficiency of terminator B014. Based on this concept, we modified Eq (5) by considering the effect of each part of the component (in the following, “part” includes terminators, RBSs, and CheY mutants that compose components). The relative diffusion rate μ_*relative*_ for each brake component can be expressed as follows:

$$\mu_{relative}\left(\text{CAL}\right)=\frac{1}{1+\text{exp}\left(a_{XB32}\left(\frac{n}{m}CAL-p\cdot b_{CheY^{**}}\right)\right)}$$ (8)

**Fig 9:**
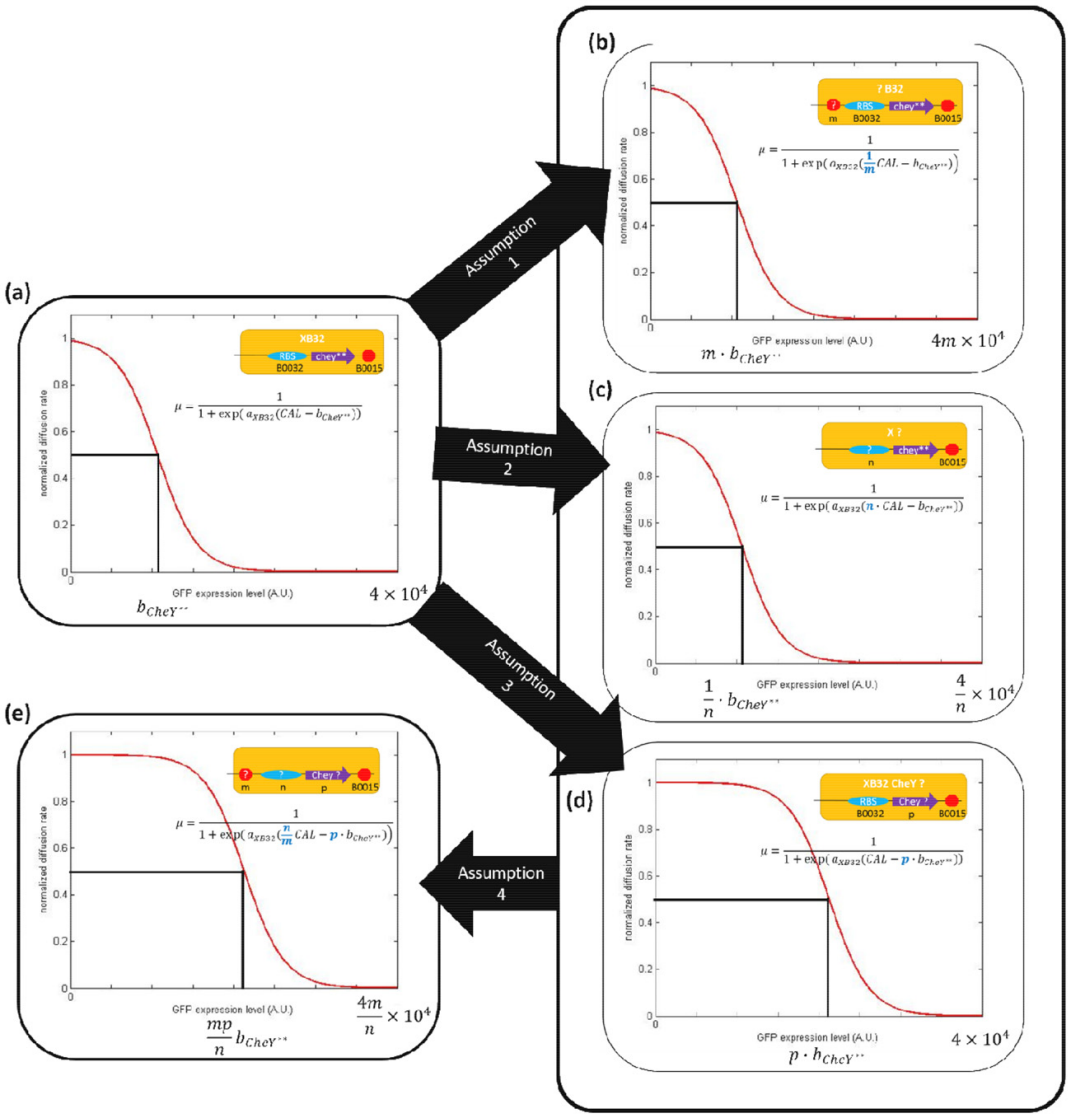
Assumptions for establishing part-based mathematical model. Component XB32 is defined as the standard component, whose composition and characteristic equation are given in (a). Assumptions 1, 2, and 3 suppose that the effect of changing a terminator, RBS or CheY mutant, can be regarded as modifying the initial characteristic equation in (a) by parameter *m*, *n*, and *p* shown in (b), (c), and (d), respectively. The modified equation can be viewed as to alter the x-axis’ scale ((b) and (c)) of the initial figure or shift the initial characteristic curve horizontally (d). Assumption 4 supposes that the effects of terminator, RBS and CheY mutant, are mutually independent. If so, every component can be represented by Eq (8) in (e) which is derived from the characteristic equation of the standard component XB32 in (a).

The equation depends on four assumptions shown in Fig 9 and listed as follows:

1. The result of a terminator can block a specific ratio of protein production. After adding a terminator, production is reduced to 1/m of the initial amount (Fig 9(b)).
2. The strength of an RBS can be represented by *n* times the strength of the RBS B0032 (Fig 9(c)).
3. *b*_CheY_**denotes CAL (the relative amount of the CheY** protein) needed to halve the bacterial population diffusion rate. For different CheY types (wild type or mutants), the CAL needed to halve the diffusion rate can be represented by *p* × *b*_CheY_** (Fig 9(d)).
4. The parameters *m*, *n*, and *p* for a component are only decided by the chosen terminator, RBS, and CheY version, respectively. The effects of the terminators, RBSs, and CheY mutants are mutually independent (Fig 9(e)).

That is, replacing a terminator (Fig 9(b)) or an RBS (Fig 9(c)) considers how to alter the scale of the x-axis of the “CAL vs. diffusion rate” curve of the standard circuit XB32 (Fig 9(a)), whereas replacing CheY** with another CheY mutant (Fig 9(d)) shifts the “CAL vs. diffusion rate” curve of the standard circuit XB32 (Fig 9(a)) horizontally. Most importantly, if the effects of all parts are mutually independent, the characteristic curve of any component can be anticipated according to its terminator, RBS and CheY mutant by Eq (8).

Further, by comparing components that differ in only one part, the parameter of that part can be obtained. Depending on this concept, the identified components in the library were compared with each other to obtain the parameters of all parts used in this study. The results are given in Table 2 as follows.

**Table 2:** Comparison of components that differ in only one part.

Comparison	Result
XB32: B14B32	*m*_B0014_ = 28.9
XB31: B14B31	*m*_B0014_ = 28.1
XB32CheYD13K: B14B32CheYD13K	*m*_B0014_ = 30.6
XB32CheY: B14B32CheY	*m*_B0014_ = 26.3
XB32: B15B32	*m*_B0015_ = 116
XB31: B15B31	*m*_B0015_ = 106.2
XB32: XB31	*n*_B0031_ = 1.0047
B14B32: B14B31	*n*_B0031_ = 1.075
B15B32: B15B31	*n*_B0031_ = 1.098
XB32: XB32CheY	*p*_*CheY*_ = 3.82
B14B32: B14B32CheY	*p*_*CheY*_ = 3.49
XB32: XB32CheYD13K	*p*_*CheY*_^*DisK*^ = 0.66
B14B32: B14B32CheYD13K	*p*_*CheY*_^*DisK*^ = 0.70

For terminators B0014 and B0015, the *m* parameters for these terminators are consistent. All the *m* parameters for B0014 and B0015 are close to 28.5 and 111, respectively. In addition, for RBS B0031, all parameters *n* are also around 1.08. This shows that the model to describe the operation of terminators and RBSs is persuasive and we can validate assumptions 1 and 2.

To verify that our model is suitable for other CheY versions, two circuits were further constructed. The component B14B32 was modified by changing its CheY** to two other CheY versions (wild type and CheY^D13K^), which yielded the components B14B32CheY and B14B32CheYD13K, respectively. These components were measured and compared to B14B32. The results are consistent with those in previous. CheY^D13K^ is the most sensitive, whereas wild type CheY is the least sensitive. Further, even if each CheY version was put with different components (XB32 or B14B32), the *p* values are quite consistent, which validate our assumption 3. The *p* values for wild type CheY and CheY^D13K^ are around 3.66 and 0.68, respectively.

From the above comparison, each part of a component (terminator, RBS, or CheY mutants) was placed in at least two different contexts. The results show that the *m*, *n*, and *p* values remain constant, supporting assumption 4 that these values are mutually independent.

**Fig 10:**
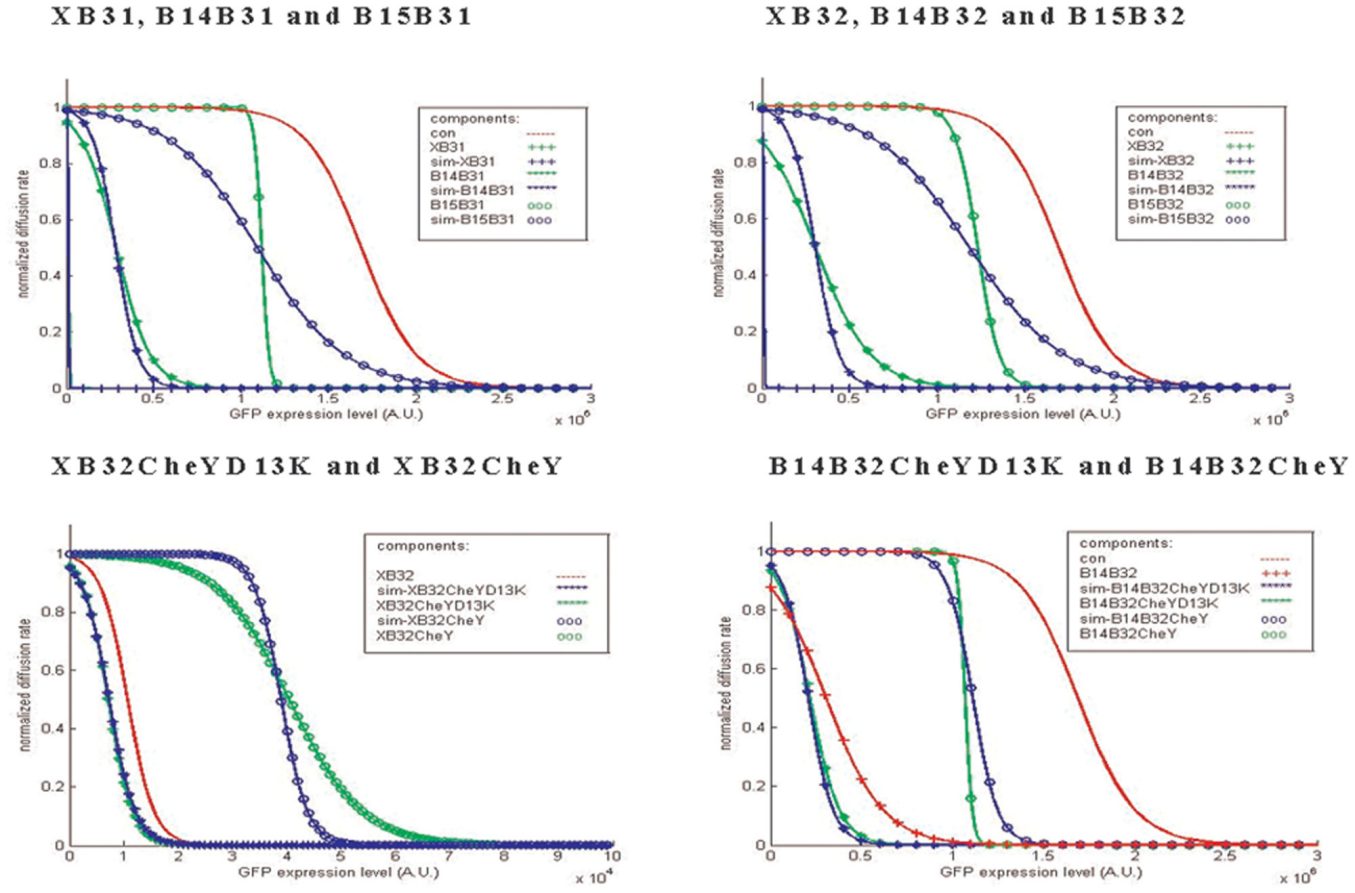
Comparison between the simulated characteristics of the components and the experimental results. The simulated characteristics of the components based on Eq (8) are shown by blue lines, whereas the experimental results of the corresponded components are shown by green lines.

Finally, in Fig 10, we simulate the characteristic of all components (blue lines) and compare them with experimental data (green lines). The results show that the model can approximately simulate the experimental results, except for components B15B31 and B15B32. The reason is that *a* values for these components deviate from α_*XB*32_. In general, α_*XB*32_ indicates how the CheY** protein influences the diffusion rate as the production of CheY** increases. A deviated *a* shows that the diffusion rate could change as the production of CheY** protein remains the same, which means there are other factors that influence the diffusion rate. Fig 7(a) shows that the diffusion rate decreases intensively at high GFP production. Thus, we consider the mass production of GFP protein that causes the diffusion rate to drop faster than expected.

In conclusion, we confirm that Eq (8) can properly depict the characteristic of the circuit depending on the composed parts. From the aforementioned comparison, the parts used in this study can be organized into a “parts library” in Table 3. Depending on this parts library, a 3 × 2 × 3 components library can be constructed without being individually verified by experiments. Further, the components library can be expanded efficiently by adding new members in the parts library.

**Table 3:** Members and parameters of the “parts library”.

Classes	Parts	Parameters
Terminator	None	m = 1
Terminator	B0014	m = 28.5
Terminator	B0015	m = 111
RBS	B0032	n = 1
RBS	B0031	n = 1.08
CheY	CheY**	p = 1
CheY	CheY wild type	p = 3.66
CheY	CheY D13K	p = 0.68

CheY**: CheY double mutant D13K Y106W.

### Design strategy for the synthetic circuit

**Fig 11:**
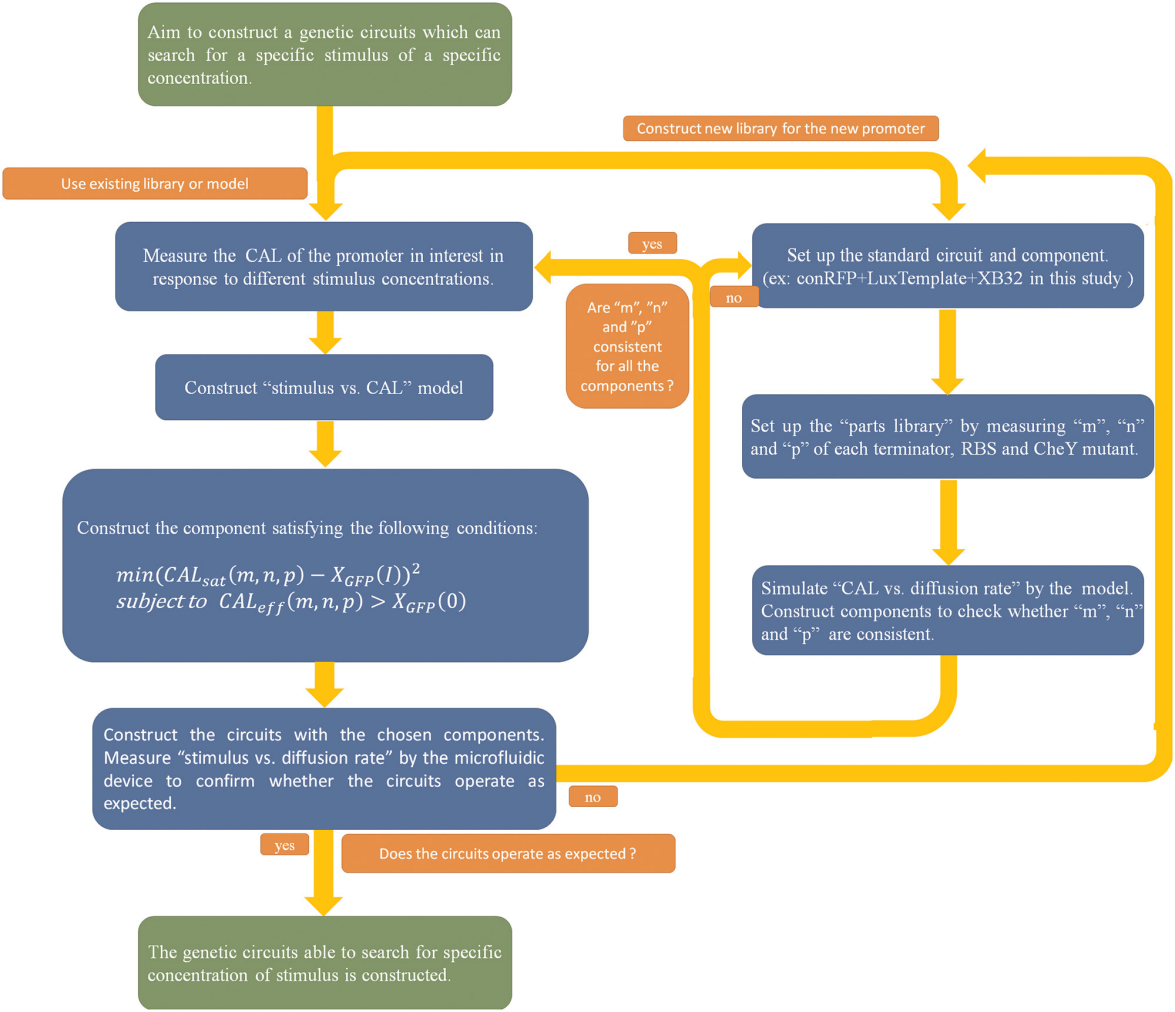
Flow chart for designing a synthetic circuit that can search for a specific stimulus of a specific concentration. This flow chart introduces the recommended procedure for designing a synthetic circuit used to search for a specific stimulus of a particular concentration. According to the flow chart, a design example is illustrated.

In this section, we propose a design procedure to construct synthetic circuits for searching for a specific concentration of stimulus by another promoter. The flow chart is shown in Fig 11.

At the beginning, one can choose to construct a new library for the new promoter (the right route) or use the existing library and model provided (the left route). In this study, components were identified according to component activation level (CAL). Despite changes in transcription efficiency due to the new promoter or environment, the relationship between CAL and the diffusion rate is likely to remain the same. Therefore, we can assume the identification of the components is still suitable for different situations.

If one chooses to use the existing library, the first task is to identify the parameters of the new promoter. We recommend that one can identify parameters using the same RBS-GFP as we did. This can avoid the coupling effect in an operon [27], and the difference in RBS at the first position may affect the efficiency of RBS at the second position. The parameter identification of the promoter provides sufficient information for establishing the “stimulus vs. CAL” model. Combined with the model in previous, the adequate component can be selected by choosing the proper parts in the “parts library” to satisfy the following minimization problem:

$$\{\begin{array}{c} min{\left(CAL_{sat}\left(m,n,p\right)-X_{GFP}\left(I\right)\right)}^{2}\\ CAL_{sat}\left(m,n,p\right)=\frac{m}{n}\left(\frac{\text{ln}\left(\frac{1-u}{u}\right)}{a_{XB32}}+p\cdot b_{CheY^{**}}\right),\;u=0.1 \end{array}$$ (9)

where *X*_*GFP*_(*I*) denotes the CAL induced by the desired concentration (I) of the stimulus, which is obtained by the “stimulus vs. CAL” model in Eq (7). Rearranging Eq (8) yields *CAL*_*sat*_, which represents the CAL at the saturation point (defined in Section 3.3 and shown in Fig 7(b)) of the component. By solving the above minimization problem, once the bacteria enter an area containing the desired stimulus concentration, the chosen brake component will attain its saturation point and deprive the bacteria of their moving ability (reduced to 10% of their initial diffusion rate).

Indeed, the effective region (shown in Fig 7(b)) of the chosen component should not cover the basal leakage of the promoter, which can be expressed as follows:

$$CAL_{eff}\left(m,n,p\right)=\frac{m}{n}\left(\frac{\text{ln}\left(\frac{1-u}{u}\right)}{a_{XB32}}+p\cdot b_{CheY^{**}}\right)>X_{GFP}\left(0\right),\;u=0.7$$ (10)

Therefore, we need to solve the constrained optimization problem in Eq (9) subject to Eq (10) from the parts library in Table 3 for the synthetic circuit. Rearranging Eq (8) yields *CAL*_*eff*_ (*m*, *n*, *p*) which represents the CAL at the cut-off of the effective region of the component. *X*_*GFP*_(0) denotes the basal leakage of the promoter, which is obtained by *X*_*GFP*_(*I*) with the stimulus concentration *I* = 0. If the component fails to meet the above condition, it will be activated consistently even if no stimulus is detected.

**Fig 12:**
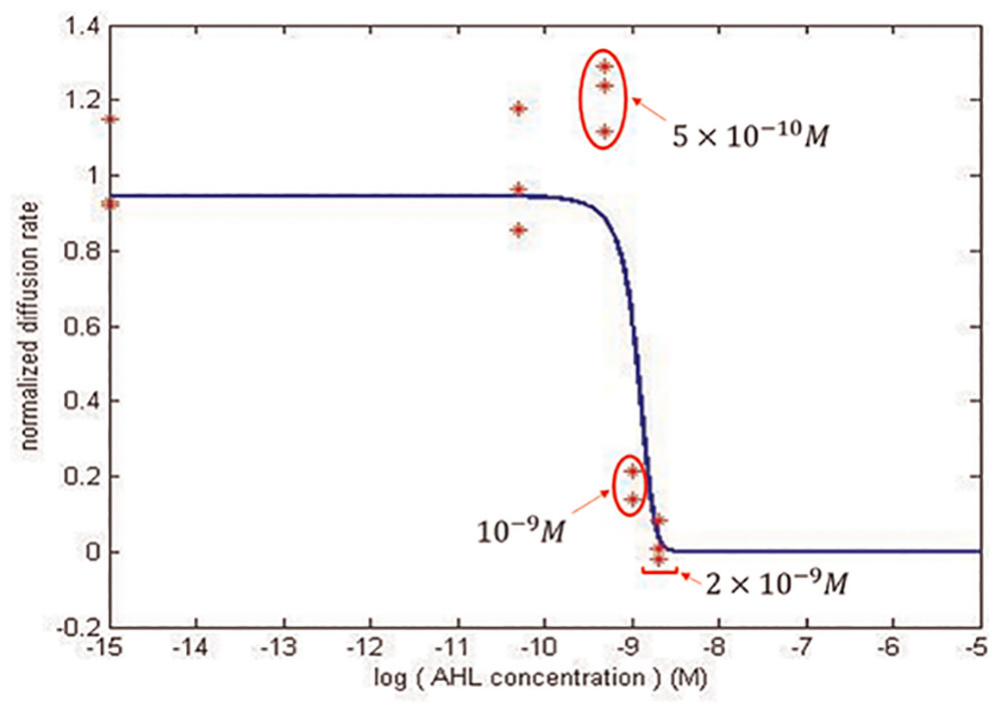
The simulated and experimental result of the component chosen to search for 10^−9^ M AHL. Following the recommended design procedure, by solving the constrained optimization Eqs (9) and (10), a standard circuit with the component B14B32CheYD13K was constructed to search for 10^−9^ M AHL. The simulated characteristic of the circuit is shown by a blue line, whereas the experimental result is shown by red stars.

To demonstrate the above design procedure, we take the case of designing a synthetic circuit to search for 10^−9^ M AHL as an example. First, the characteristics of the plux promoter system have to be identified by experiment to establish the mathematical model for the promoter system. The result is given in Fig C in S1 File. Based on this information, for all the components, the bacterial population diffusion rate at all AHL concentrations can be simulated, as shown in Fig 8(c). Further, the adequate component can be composed by choosing the proper parts of the “parts library” in Table 3 to satisfy the minimization problem in Eq (9). After solving the constrained optimization problem in (9) and (10), the component B14B32CheYD13K is the most effective component for the synthetic circuit. For B14B32CheYD13K, *CAL*_*sat*_ = 3.6 × 10^5^, which is close to *X*_*GFP*_(10^−9^) = 1.7 × 105, and *CAL*_*eff*_ = 1.5 × 10^5^ > *X*_*GFP*_(0) = 2.7 × 10^3^. Therefore, the component B14B32CheYD13K is a good choice for constructing the synthetic circuit. Finally, the characteristic of the circuit were measured and verified in different AHL concentrations. The result is given in Fig 12 to confirm that bacteria almost stop as the concentration of AHL rises to 10^−9^ M.

After selecting the proper components, one may construct the complete circuits and observe the relationship between stimulus concentration and the bacterial population diffusion rate by microfluidic device quantitatively or simply by microscope qualitatively. If the circuits operate as expected, the genetic circuits that are able to search for a specific concentration of stimulus can be constructed. However, we cannot guarantee that the components we identified operate the same in different environments or bacteria. In these cases, a new components library has to be established.

Utilizing the model, one can construct a new component library efficiently with the following procedure. First, set up the standard circuit and component (ex: conRFP + LuxTemplate + XB32). Second, construct the “parts library” by measuring *m*, *n*, and *p* of each terminator, RBS, and CheY versions. Finally, simulate “CAL vs. diffusion rate” by the model and construct the components to ensure *m*, *n*, and *p* are consistent. If not, one can try another standard circuit composition or choose other parts. Following these steps, the library can be established efficiently. For example, one can construct a component library containing 64 components using 4 terminators, 4 RBSs, and 4 CheY mutants. Further, by the comparison method, only 10 components (1 standard component, 3 for measuring terminators, 3 for measuring RBSs, and 3 for measuring CheY mutants) must be identified to obtain all *m*, *n*, and *p* values. Once the library is constructed, one can design the synthetic circuits following the above mentioned procedure (the left route).

## Discussion

Compared with previous studies, we improved the searching efficiency by adopting a search-then-locate strategy rather than the stop-then-trace strategy [9, 10]. Proven by the swarm assays and microfluidic devices, *E*. *coli* show sufficient mobility to navigate the environment until they detect adequate amounts of AHL. Further, we modified our circuits design to create components with various sensitivities. These components can be chosen to make bacteria search for specific concentrations of molecules. This solved the problem that the search-then-locate strategy has a limited searching range. That is, if the molecule is too dense, the bacterial population will stop far away from the source. By choosing the appropriate components for systematic circuits, we can not only solve this limited searching range problem, but also provide other applications. For example, the bacteria can be controlled so as to not approach specific areas or other bacterial populations by choosing considerably different sensitivity components.

The components were verified qualitatively by swarm assays and quantitatively by microfluidic devices. The swarm assay was originally adopted for quantifying the diffusion rate in this study. However, the chemotaxis phenomenon of the strain RP437 complicates the diffusion calculation. As a result, we selected the strain RP5232, a non-chemotactic smooth swimming strain, for the swarm assay. Nevertheless, the strain was immobile in the semi-solid agar because smooth swimming makes *E*. *coli* susceptible to becoming trapped in agar. Previous studies have shown that bacteria spread effectively in agar when tumbles are frequent. However, the bacteria that tumble incessantly also fail to spread effectively [28]. The swarm assay is not suitable for measuring diffusion rate in this study due to diffusion rate lacking a clear relationship with tumbling in agar, where tumbling is the main factor influencing diffusion.

As a result, a microfluidic device is used to measure diffusion rate. RP5232 is selected to carry the synthetic circuits because of its non-chemotactic properties. Without being influenced by the chemotaxis phenomenon, the main factor that influences bacterial population diffusion is the amount of CheY** protein. To simplify the model, the model in Eq (5) is directly connected with the relative amount of CheY** (CAL) and the corresponding diffusion rate. The complicated relationships among the amount of CheY**, tumbling frequency, and diffusion rate are neglected because they have already been discussed in previous studies [29, 30, 31, 32]. Moreover, the simplified model is still confirmed by experimental result.

Based on this model, the efficiencies of all parts (terminators, RBS, wild type CheY, and CheY mutant) used in the components were calculated. Compared to previous studies, the efficiencies of these parts are not consistent. For example, the terminator B0014 and B0015 in a previous study have a termination efficiency of 60.4% and 98.4%, respectively [17]. However, the efficiencies (calculated by 1-1/m) of B0014 and B0015 are 96.5% and 99.1% in this study. Another previous study [16] shows that RBS B0032 is stronger than B0031; in this study, B0031 is slightly stronger than B0031 (*n*_*B*0031_ = 1.08), suggesting the efficiency of these parts depends significantly on the context. That is, the identification of the parts should be measured and compared with complete circuits, as we did. Identifying each part separately is not recommended for establishing the “parts library” in this study.

## Conclusion

In this study, we engineered synthetic circuits for bacteria to search for specific molecules using a systematic synthetic biology method. Under the control of the synthetic circuits, the bacteria can navigate the environment until they detect specific molecules and stop at a particular concentration in the environment. Particularly, components library and mathematical models were constructed to facilitate the systematic design of synthetic circuits that prompt bacteria to search for a specific concentration of molecule. These engineered bacteria may be applied to many situations, such as searching out and destroying herbicides [3] or invading cancer cells [4]. Further, we anticipate that controlling the spatial distribution of the bacteria population is beneficial for creating bacteria consortia, which can cooperate efficiently. Well-cooperating and engineered consortia have the potential for carrying out complex functions normally unachievable for single strains. We hope that our systematic design method can expand the field of synthetic biology and boost tangible advances in synthetic biotechnology.

## Supporting Information

S1 File Supplementary information. (PDF)
