# Supplementary material for: Engineering Bacteria to Search for Specific Concentrations of Molecules by a Systematic Synthetic Biology Design Method
Source: PLoS One. 2016 Apr 20;11(4):e0152146. doi: 10.1371/journal.pone.0152146 (PMC4838244; doi:10.1371/journal.pone.0152146)
Supplement: S1 File — (PDF) [file pone.0152146.s001.pdf]

Supplementary Information

A. Supplement Tables

Table A. Primers used in this study

Gene or Mutant	Primer sequences
cheY	Forward:<br>TATGAATTCGCGGCCGCTTCTAGAATGGCGGATAAAGAA<br>Reverse:<br>ATACTGCAGCGGCCGCTACTAGTTCACATGCCCAGTTT
D13K	Forward:<br>TTTTTGGTTGTGGATAAATTTTCCACCATGCGA<br>Reverse:<br>TCGCATGGTGGAAAATTTATCCACAACCAAAAA
Y106W	Forward:<br>CGGGGGCCAGTGGCTGGGTGGTGAAGCCATTTA<br>Reverse:<br>TAAATGGCTTCACCACCCAGCCACTGGCCCCCG

Table B. Sequences information

Promoters	Constitutive Promoters	
	BBa_J23101	tttacagctagctcagtcctaggtattatgctagc
	BBa_J23105	tttacggctagctcagtcctaggtactatgctagc
	Activator-regulated Promoter (LuxR & HSL regulated promoter)	
	BBa_R0062	acctgtaggatcgtacaggtttacgcaagaaaatggtttgttatagtcgaataaa
	Repressor-regulated Promoter (TetR repressible promoter)	
	BBa_R0040	tccctatcagtgatagagattgacatccctatcagtgatagagatactgagcac
RBS	Ribosome Binding Sites	
	BBa_B0031	tcacacaggaaacc
	BBa_B0032	tcacacaggaaag
	BBa_B0034	aaagaggagaaa

Terminators	Terminators	
	BBa_B0014	tcacactggctcaccttcgggtgggcctttctgcgtttatatactagagagagaatataaaaagccagattattaatccggcttttttattattt
	BBa_B0015	Ccaggcatcaaataaaacgaaaggctcagtcgaaagactgggcctttcgttttatctgttgtttgtcggtgaacgctctctactagagtcacactggctcaccttcgggtgggcctttctgcgtttata
Regulator Proteins	LuxR Activator	
	BBa_C0062	Atgaaaaacataaatgccgacgacacatacagaataattaataaaattaaagcttgtagaagcaataatgatattaatcaatgcttatctgatatgactaaaatggtacattgtgaatattatttactcgcgatcatttatcctcattctatggttaaatctgatatttcaatcctagataattaccctaaaaaatggaggcaatattatgatgacgctaatttaataaaatatgatcctatagtagattattctaactccaatcattcaccaattaattggaatatatttgaaaacaatgctgtaaataaaaaatctccaaatgtaattaaagaagcgaaaacatcaggtcttatcactgggtttagtttccctattcatacggctaacaatggcttcggaatgcttagttttgcacattcagaaaaagacaactatatagatagtttatttttacatgcgtgtatgaacataccattaattgttccttctctagttgataattatcgaaaaataaatatagcaaataataaatcaaacaacgatttaaccaaaagagaaaaagaatgtttagcgtgggcatgcgaaggaaaaagctcttgggatatttcaaaaatattaggttgcagtgagcgtactgtcactttccatttaaccaatgcgcaaatgaaactcaatacaacaaaccgctgccaaagtatttctaaagcaattttaacaggagcaattgattgcccatactttaaaaattaataacactgatagtgctagtgtagatcac
Reporter Proteins	Green Fluorescent Protein	
	BBa_E0040	Atgcgtaaaggagaagaacttttcactggagttgtcccaattcttgttgaattagatggtgatgttaatgggcacaaattttctgtcagtggagagggtgaaggtgatgcaacatacggaaaacttacccttaaatttatttgcactactggaaaactacctgttccatggccaacacttgtcactactttcggttatggtgttcaatgctttgcgagatacccagatcatatgaaacagcatgactttttcaagagtgccatgcccgaaggttatgtacaggaaagaactatatttttcaaagatgacgggaactacaagacacgtgctgaagtcaagtttgaaggtgatacccttgttaatagaatcgagttaaaaggtattgattttaaagaagatggaaacattcttggacacaaattggaatacaactataactcacacaatgtatacatcatggcagacaaacaaaagaatggaatcaaagttaacttcaaaattagacacaacattgaagatggaagcgttcaactagcagaccattatcaacaaaatactccaattggcgatggccctgtccttttaccagacaaccattacctgtccacacaatctgccctttcgaaagatcccaacgaaaagagagaccacatggtccttcttgagtttgtaacagctgctgggattacacatggcatggatgaactatacaaataataa
	Red Fluorescent Protein	

	BBa_E1010	Atggcttcctccgaagacgttatcaaagagttcatgcgtttcaaagttcgtatggaaggttccgttaacggtcacgagttcgaaatcgaaggtgaaggtgaaggtcgtccgtacgaaggtacccagaccgctaaactgaaagttaccaaaggtggtccgctgccgttcgcttgggacatcctgtccccgcagttccagtacggttccaaagcttacgttaaacacccggctgacatcccggactacctgaaactgtccttcccggaaggtttcaaatgggaacgtgttatgaacttcgaagacggtggtgttgttaccgttacccaggactcctccctgcaagacggtgagttcatctacaaagttaaactgcgtggtaccaacttcccgtccgacggtccggttatgcagaaaaaaaccatgggttgggaagcttccaccgaacgtatgtacccggaagacggtgctctgaaaggtgaaatcaaaatgcgtctgaaactgaaagacggtggtcactacgacgctgaagttaaaaccacctacatggctaaaaaaccggttcagctgccgggtgcttacaaaaccgacatcaaactggacatcacctcccacaacgaagactacaccatcgttgaacagtacgaacgtgctgaaggtcgtcactccaccggtgcttaataacgctgatagtgctagtgtagatcgc
Other Functional Proteins	cheY	
	cheY	Atggcggataaagaacttaaatttttggttgtggatgacttttccaccatgcgacgcatagtgcgtaacctgctgaaagagctgggattcaataatgttgaggaagcggaagatggcgtcgacgctctcaataagttgcaggcaggcggttatggatttgttatctccgactggaacatgcccaatatggatggcctggaattgctgaaaacaattcgtgcggatggcgcgatgtcggcattgccagtgttaatggtgactgcagaagcgaagaaagagaacatcattgctgcggcgcaagcgggggccagtggctatgtggtgaagccatttaccgccgcgacgctggaggaaaaactcaacaaaatctttgagaaactgggcatgtga
	cheY D13K (cheY*)	
	cheY*	atggcggataaagaacttaaatttttggttgtggataaattttccaccatgcgacgcatagtgcgtaacctgctgaaagagctgggattcaataatgttgaggaagcggaagatggcgtcgacgctctcaataagttgcaggcaggcggttatggatttgttatctccgactggaacatgcccaatatggatggcctggaattgctgaaaacaattcgtgcggatggcgcgatgtcggcattgccagtgttaatggtgactgcagaagcgaagaaagagaacatcattgctgcggcgcaagcgggggccagtggctatgtggtgaagccatttaccgccgcgacgctggaggaaaaactcaacaaaatctttgagaaactgggcatgtga
	cheY D13K Y106W (cheY**)	
	cheY**	atggcggataaagaacttaaatttttggttgtggataaattttccaccatgcgacgcatagtgcgtaacctgctgaaagagctgggattcaataatgttgaggaagcggaagatggcgtcgacgctctcaataagttgcaggcaggcggttatggatttgttatctccgactggaacatgcccaatatggatggcctggaattgctgaaaacaattcgtgcggatggcgcgatgtcggcattgccagtgttaatggtgactgcagaagcgaagaaagagaacatcattgctgcggcgcaagcgggggccagtggctgggtggtgaagccatttaccgccgcgacgctggaggaaaaactcaacaaaatctttgagaaactgggcatgtga

B. Supplementary Figures

Fig A. Results of the bacteria population diffusion rate measured by the microfluidic device.

Figure S1 shows all the bacterial diffusion rate experiments in this study. Several AHL concentrations were tested for each component. The calculated values of $\mu(t + t_0)$ over time are shown in the figure to obtain the diffusion rate μ by linear regression (detailed method is discussed in Section 3.1). For each component, the average of the diffusion rate at each AHL concentration is summarized in the last figure and compared with the result of the "con" component. All the components tested in this study are listed.

(a) con
(b) XB31
(c) XB32
(d) B14B31
(e) B14B32
(f) B15B31
(g) B15B32
(h) XB32CheY
(i) B14B32CheY
(j) XB32CheY D13K
(k) B14B32CheY D13K

(a) Standard circuit +“con” component

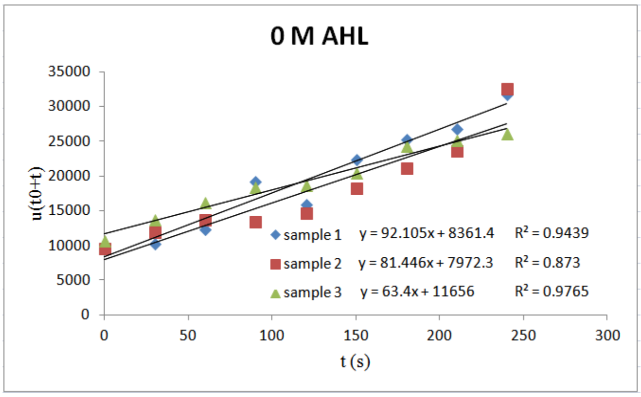

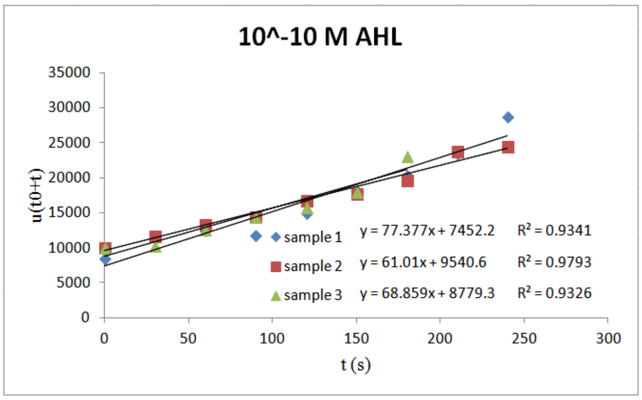

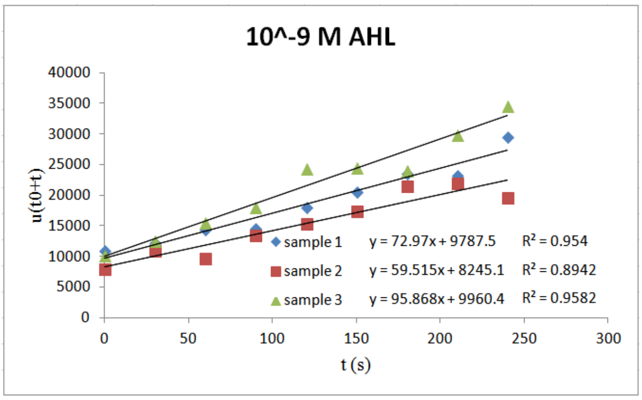

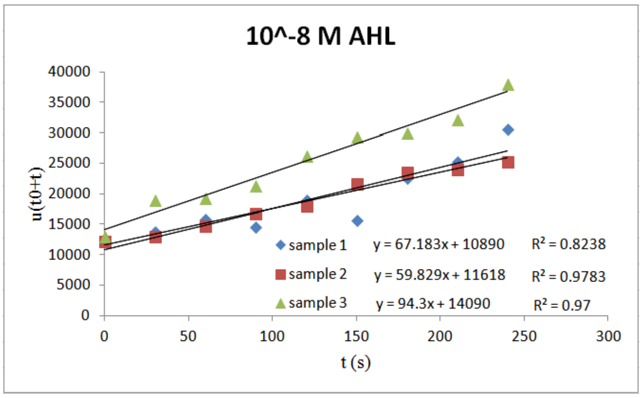

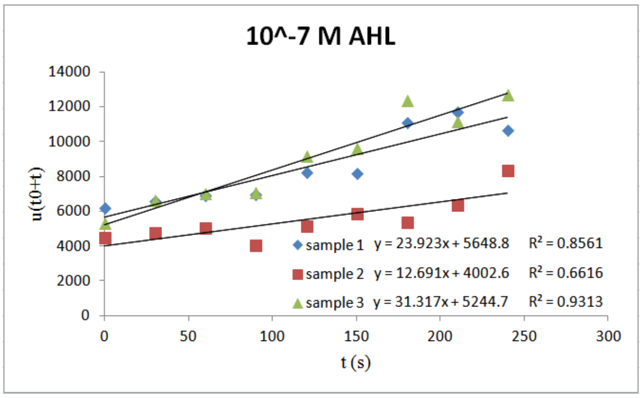

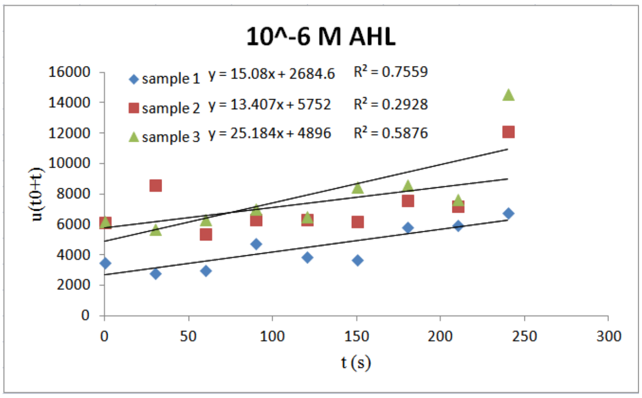

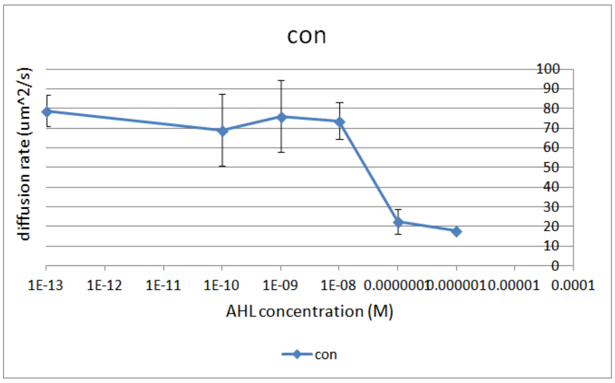

(b) Standard circuit +”XB31” component

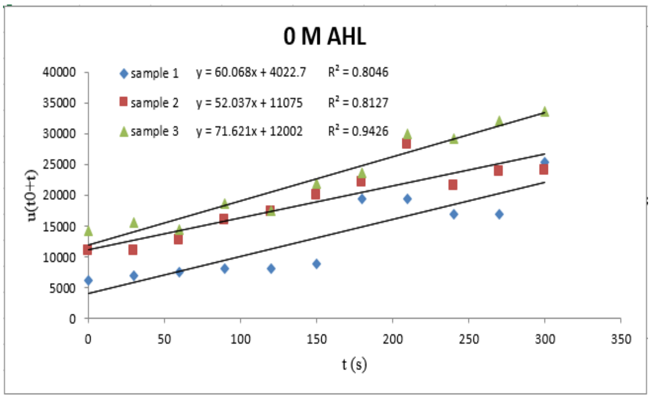

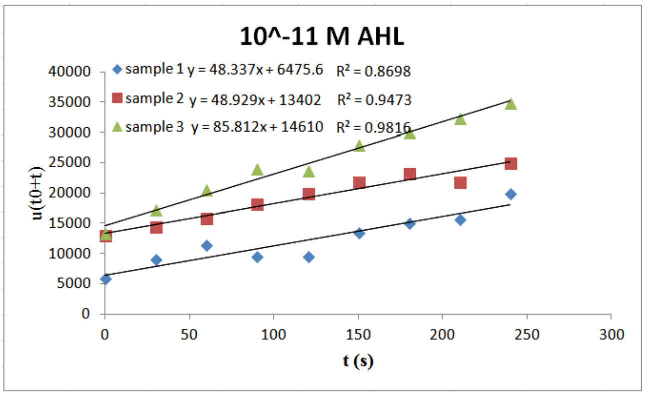

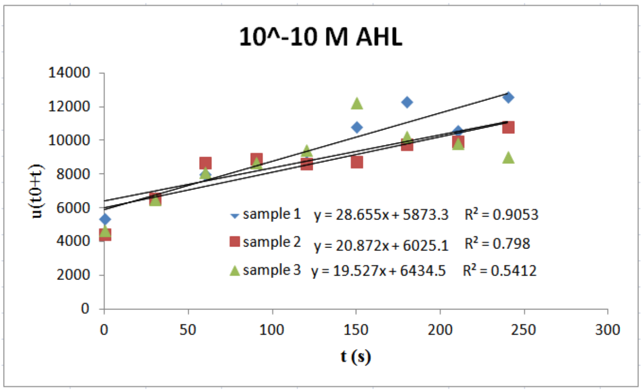

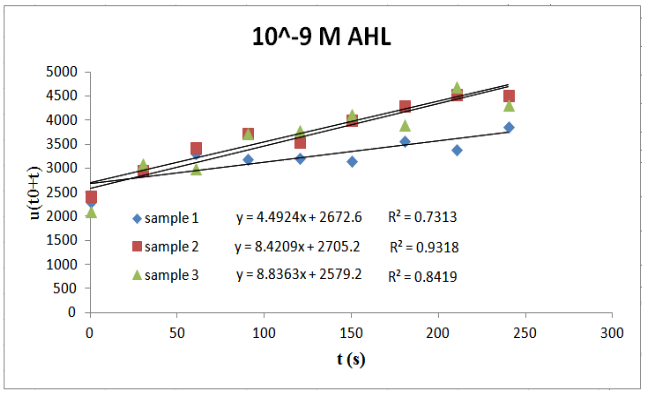

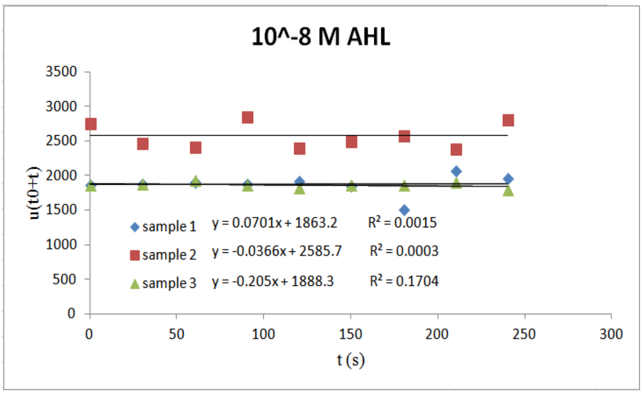

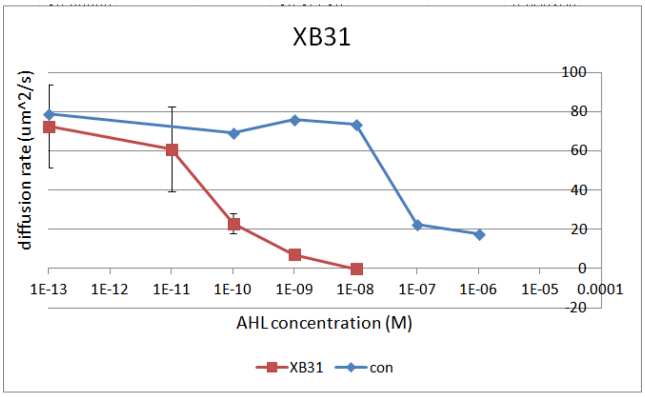

(c) Standard circuit +"XB32" component

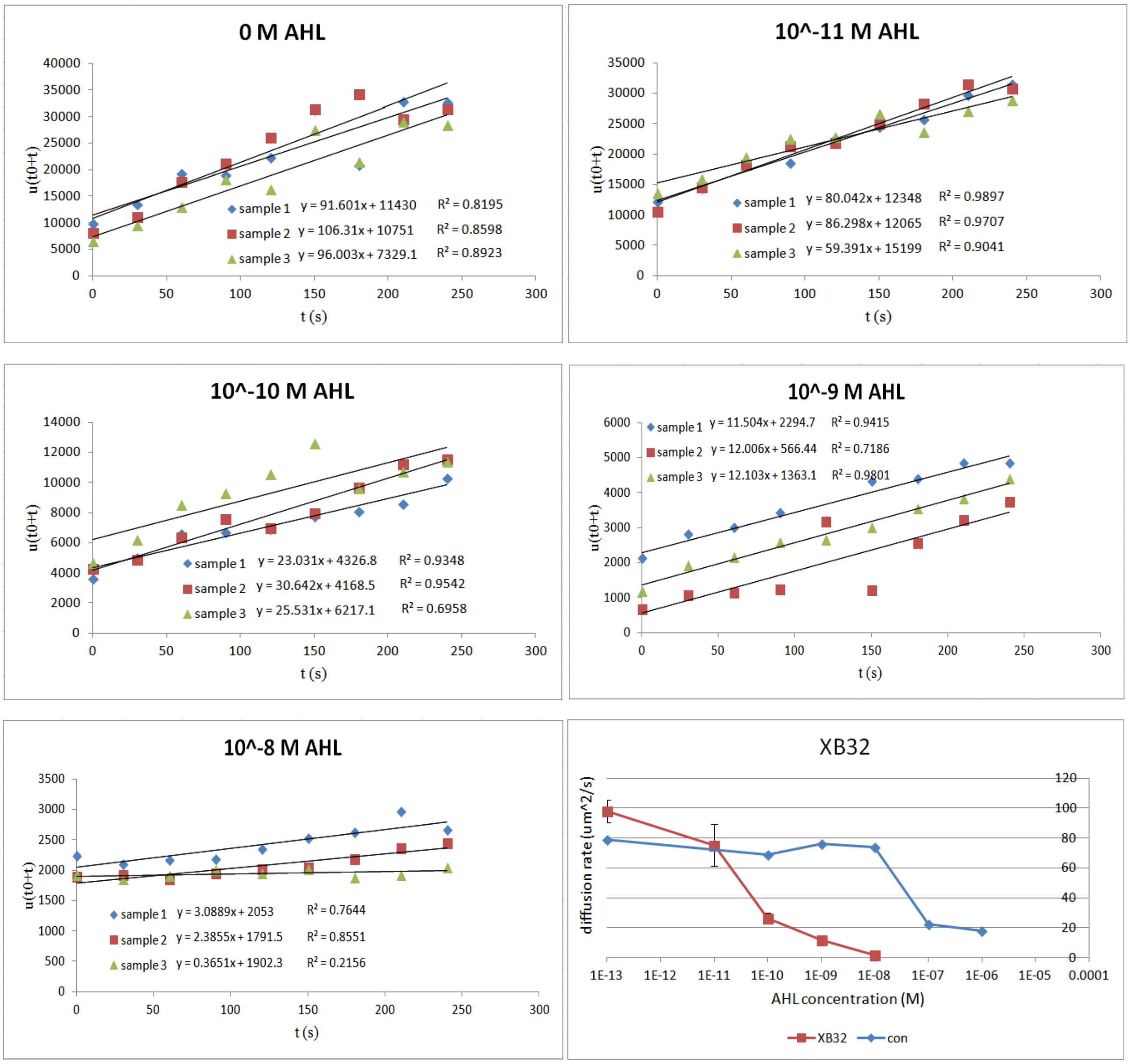

(d) Standard circuit +”B14B31” component

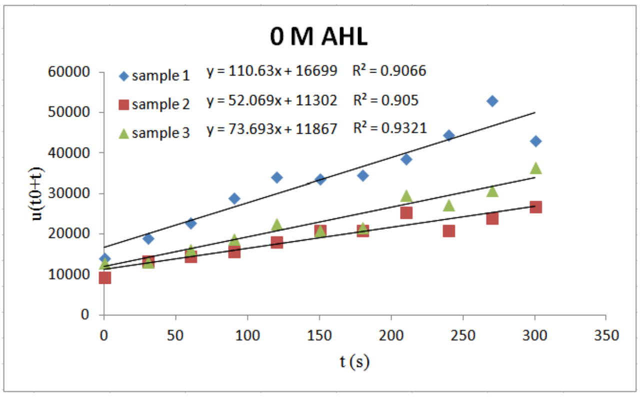

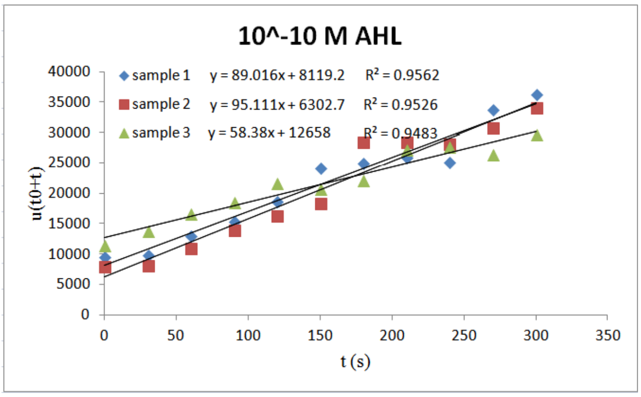

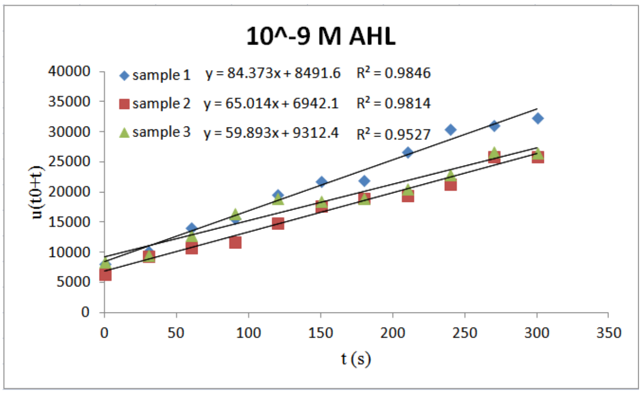

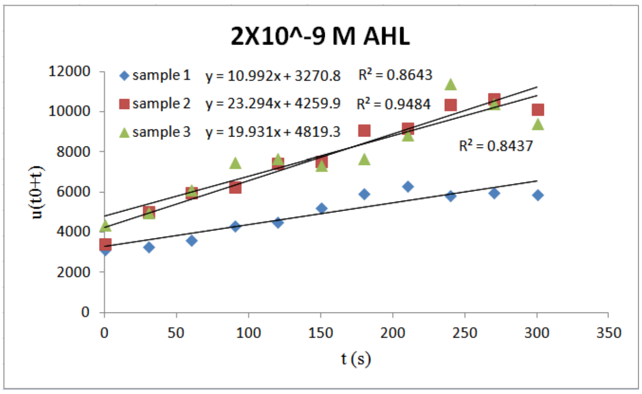

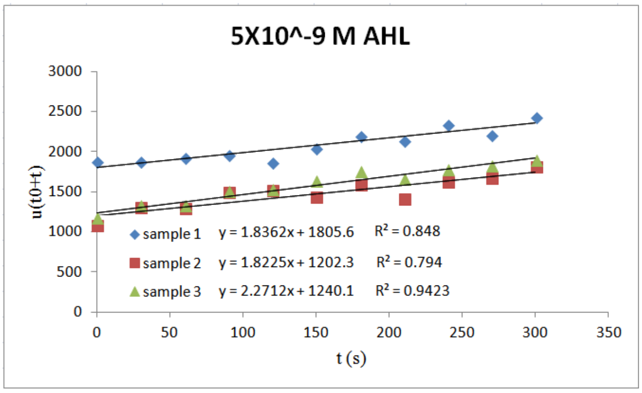

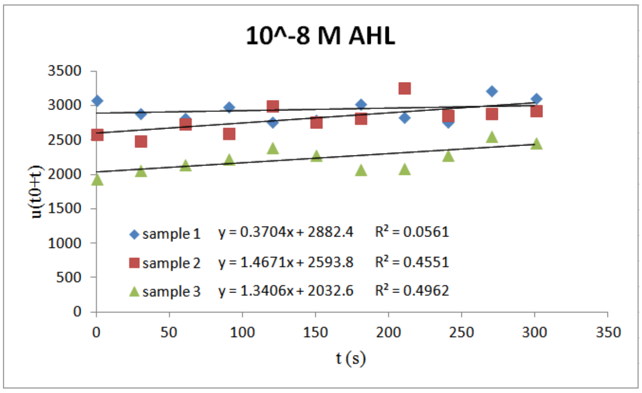

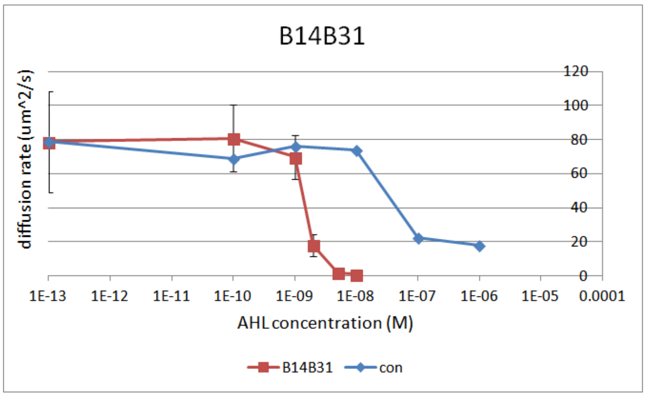

(e) Standard circuit + “B14B32” component

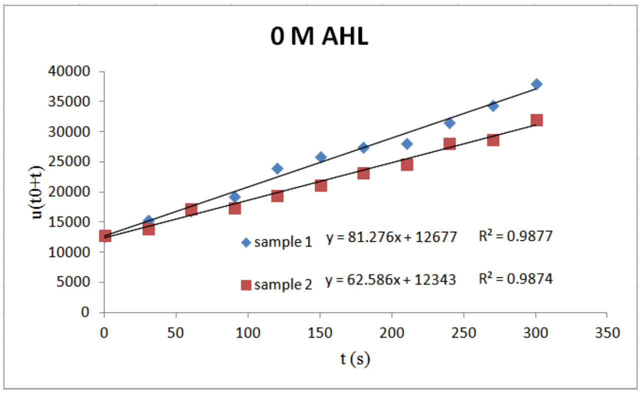

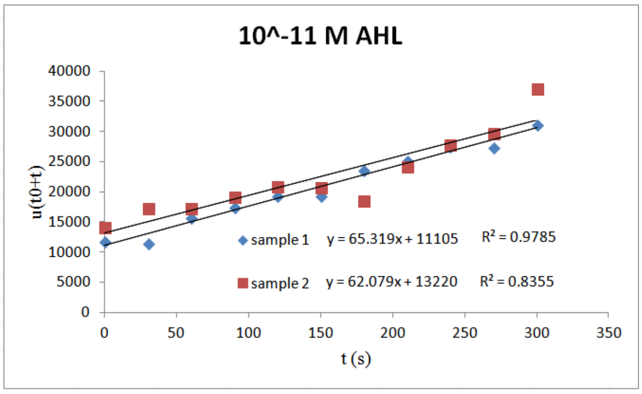

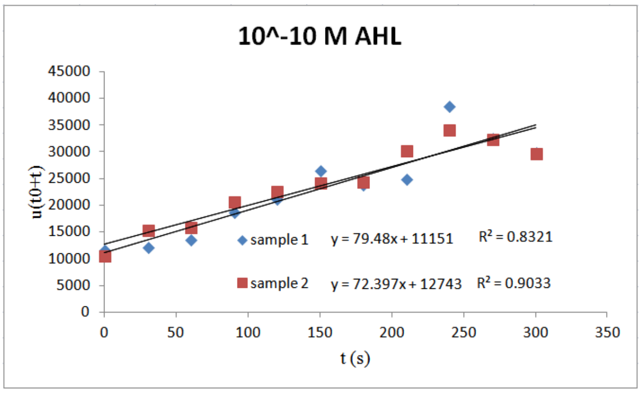

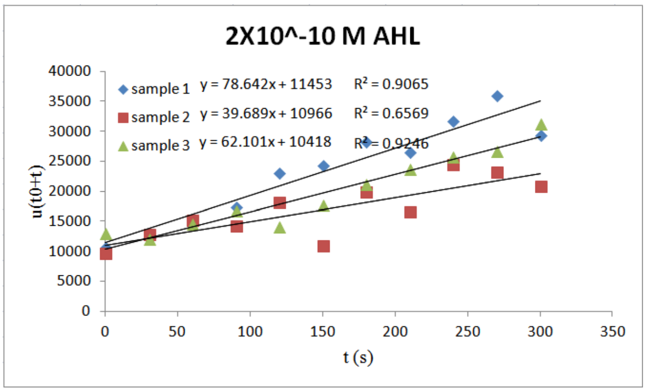

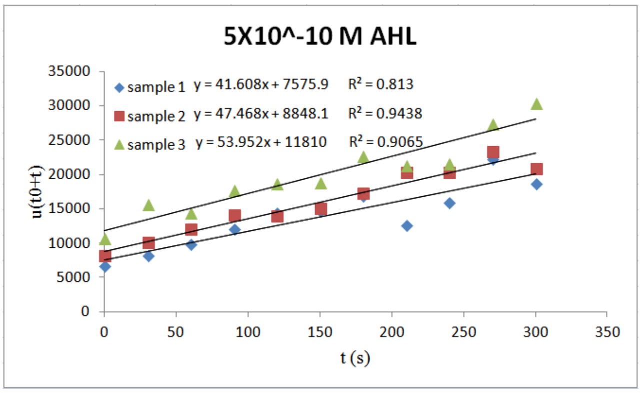

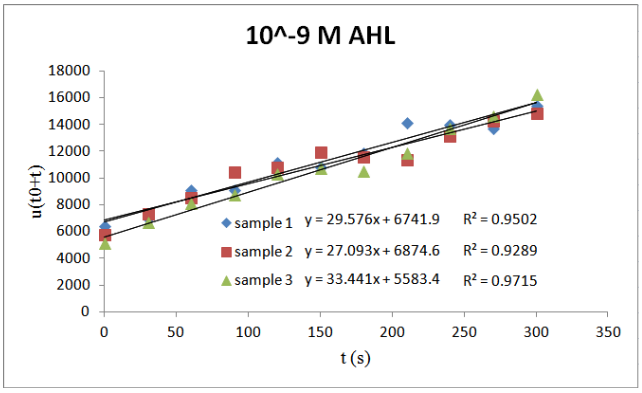

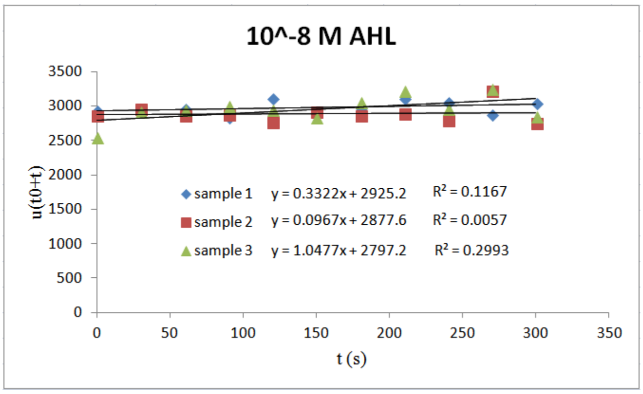

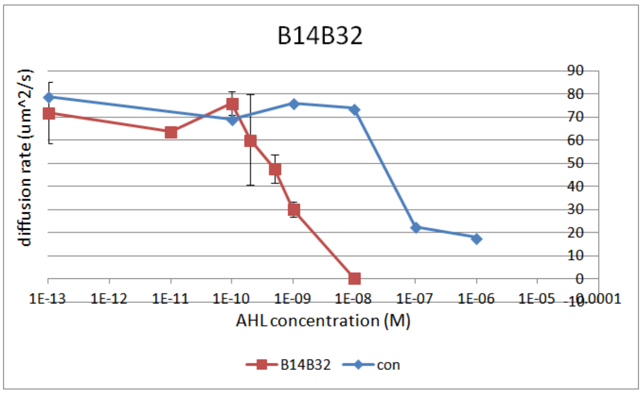

(f) Standard circuit +"B15B31" component

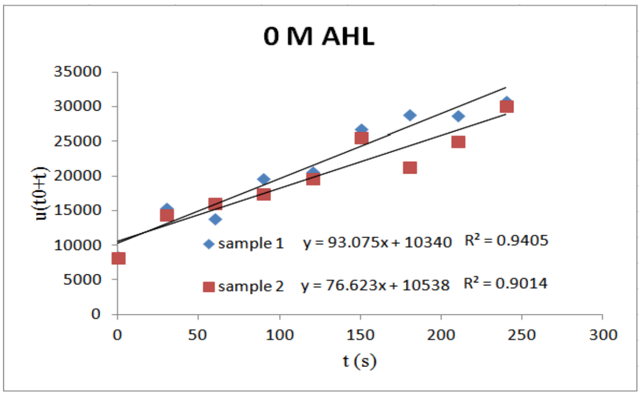

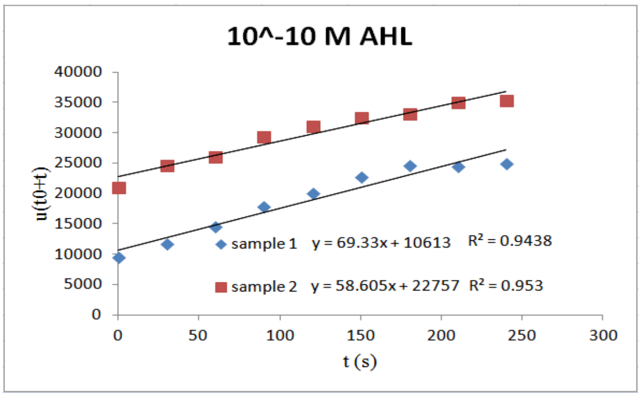

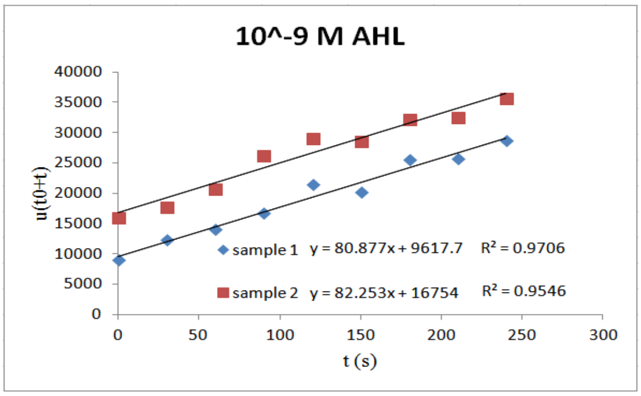

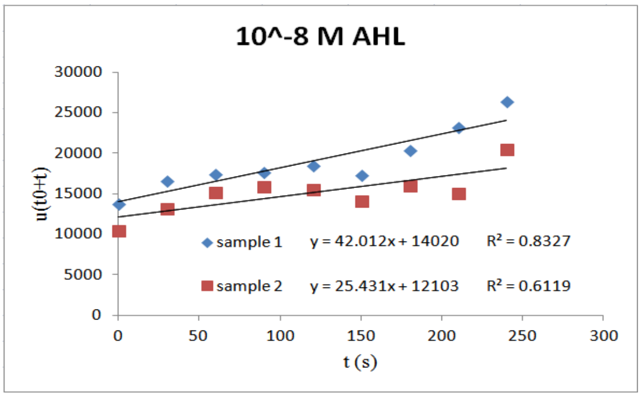

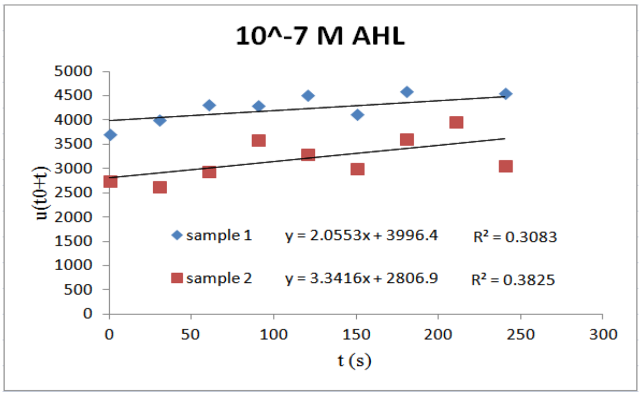

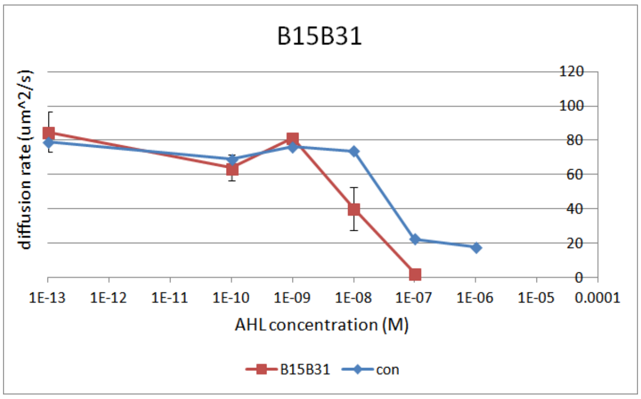

(g) Standard circuit +”B15B32” component

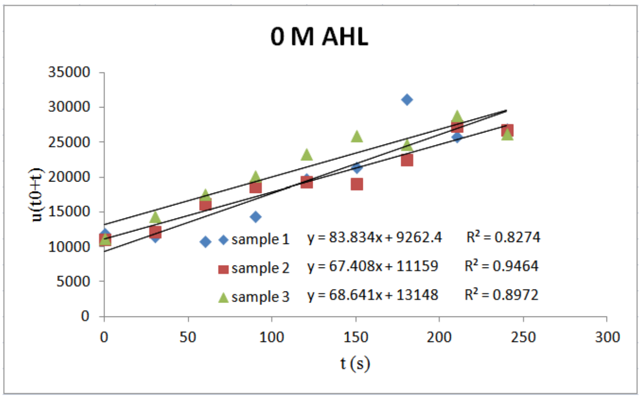

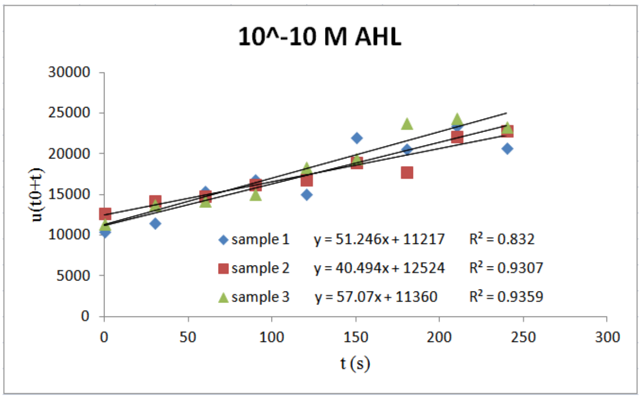

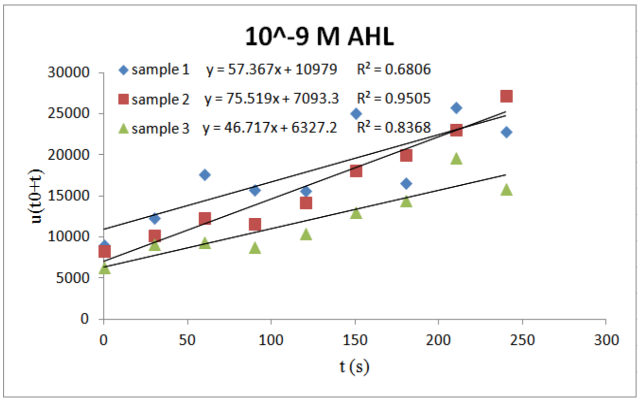

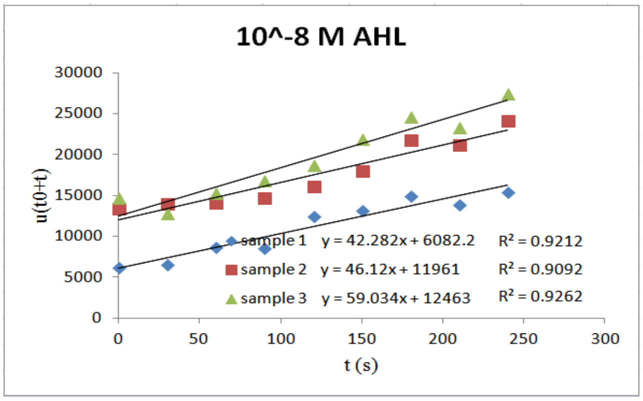

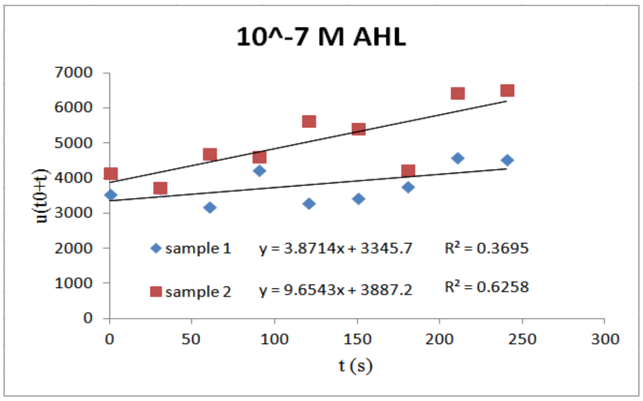

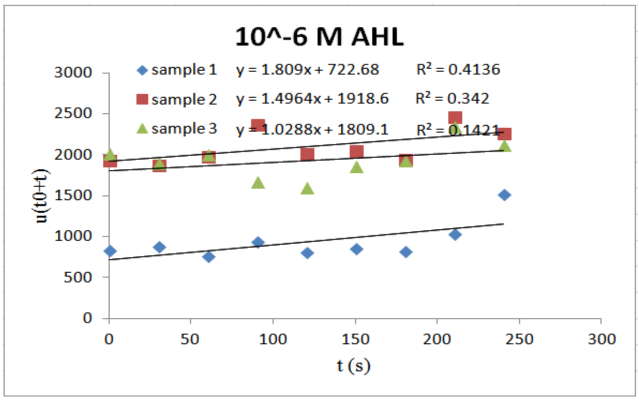

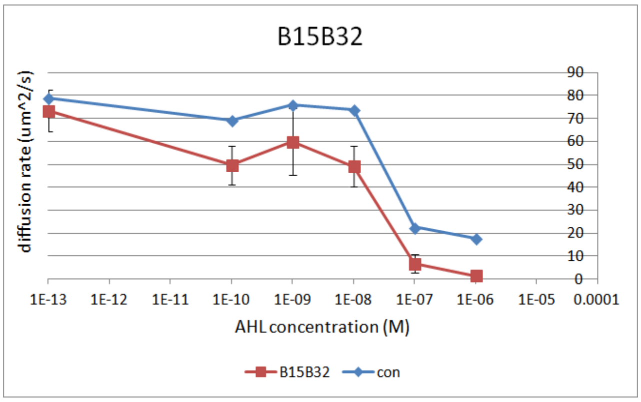

(h) Standard circuit + "XB32CheY" component

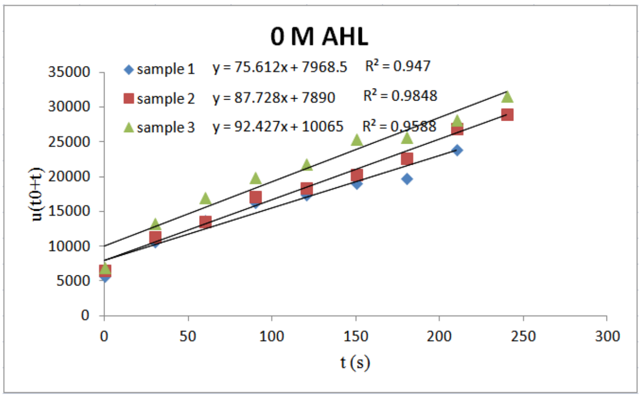

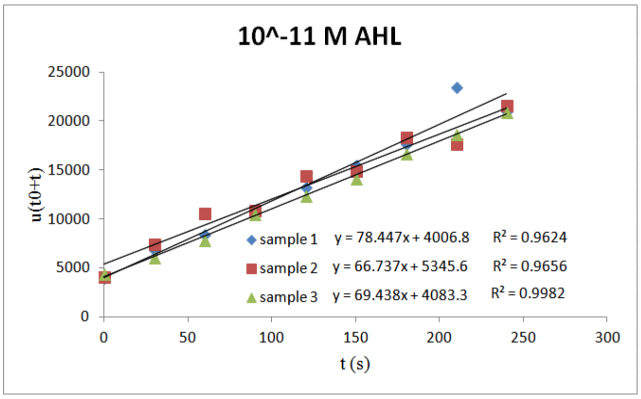

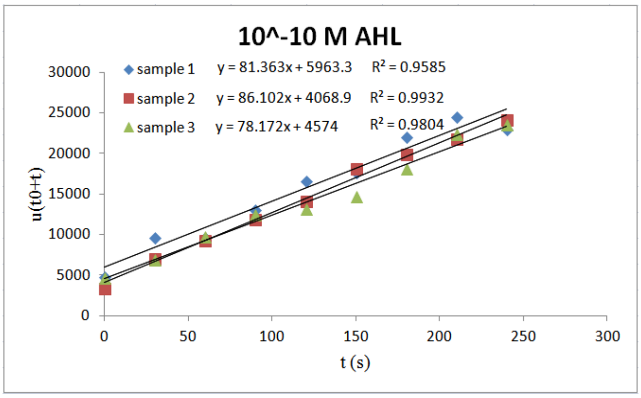

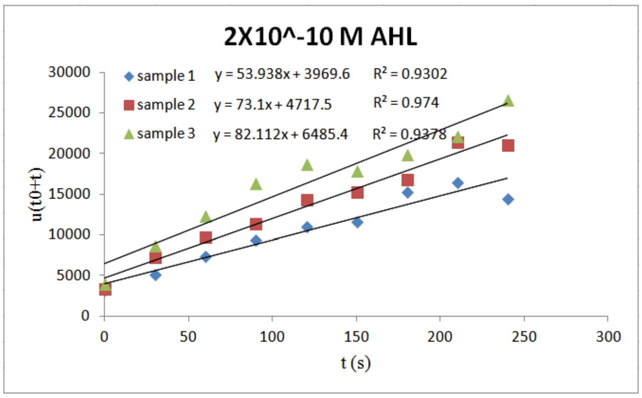

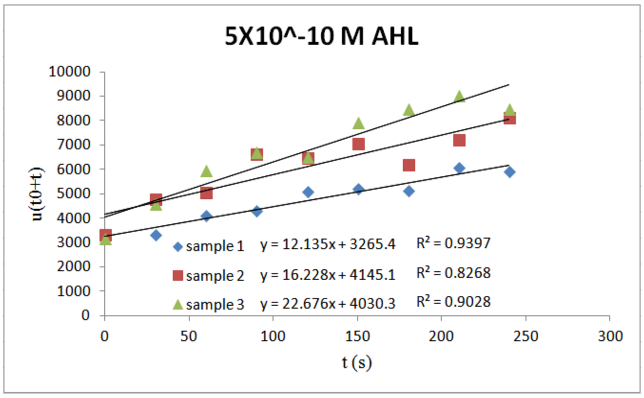

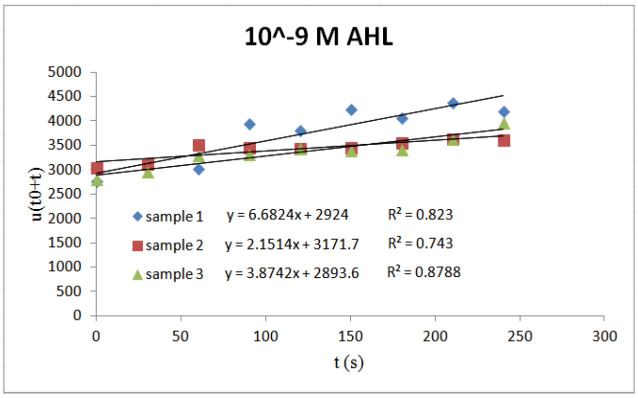

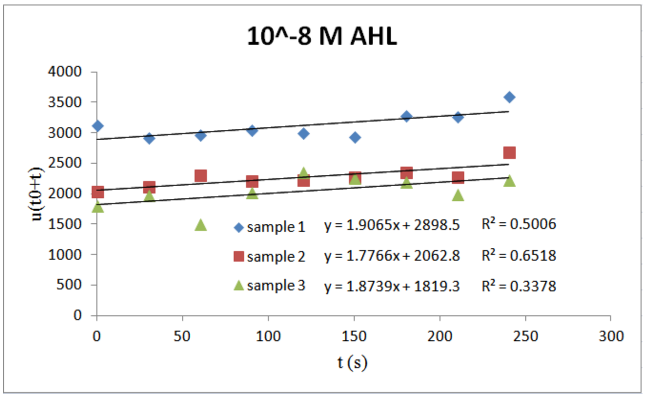

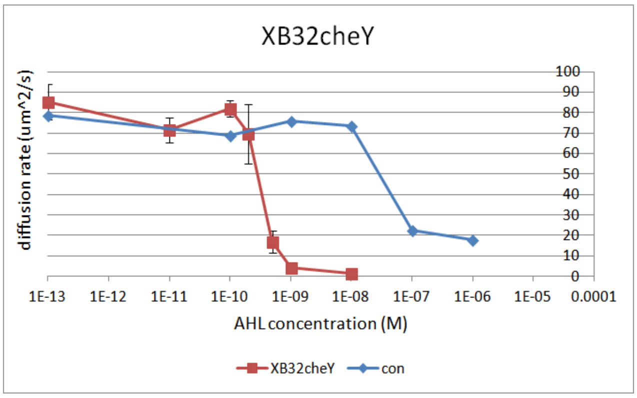

(i) Standard circuit + “B14B32CheY” component

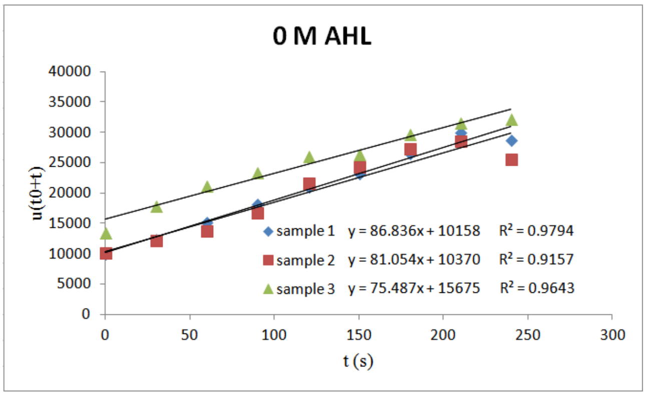

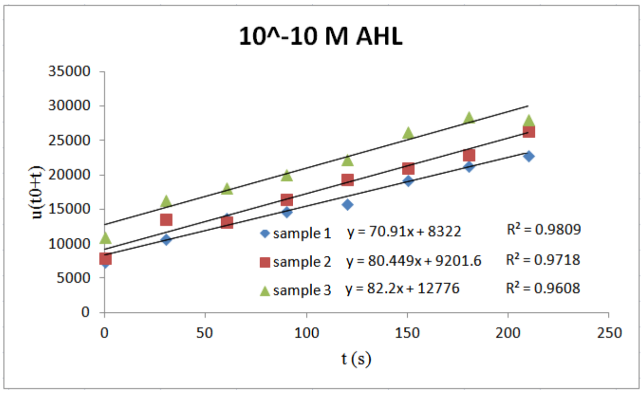

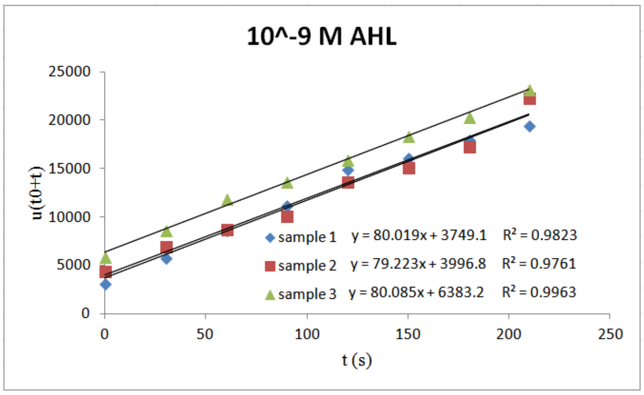

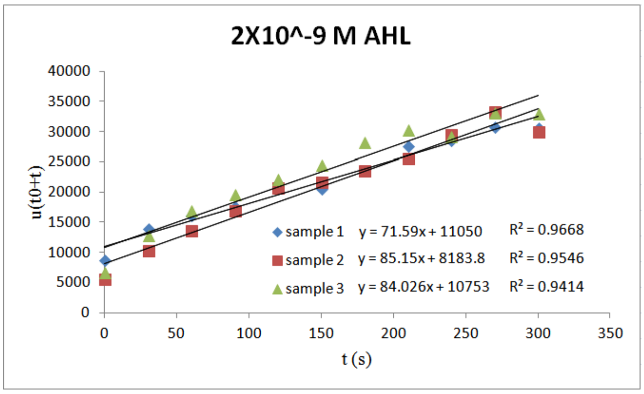

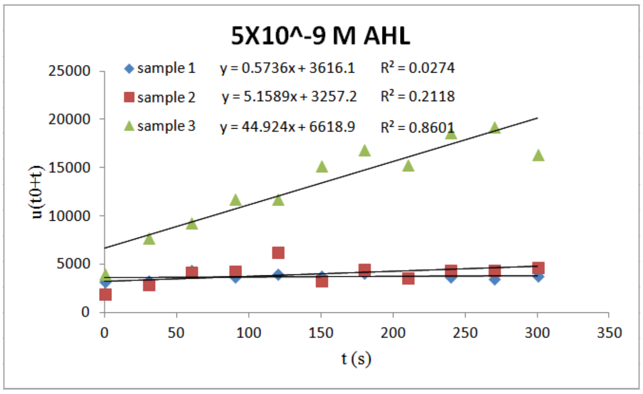

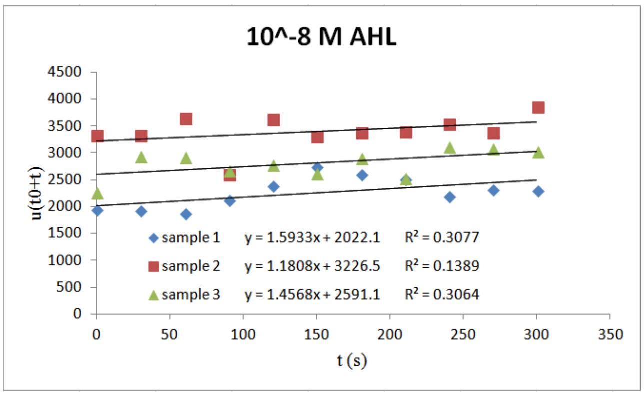

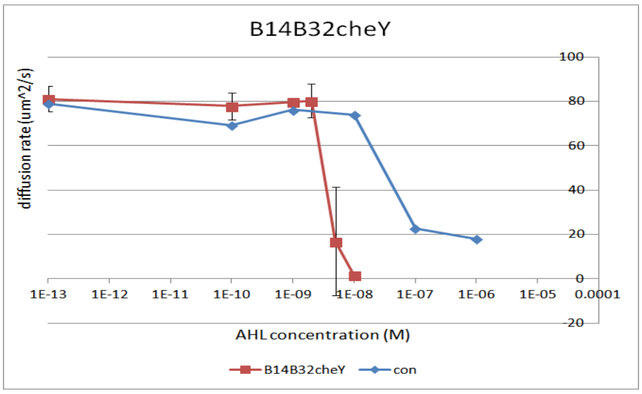

(j) Standard circuit + “XB32CheY D13K” component

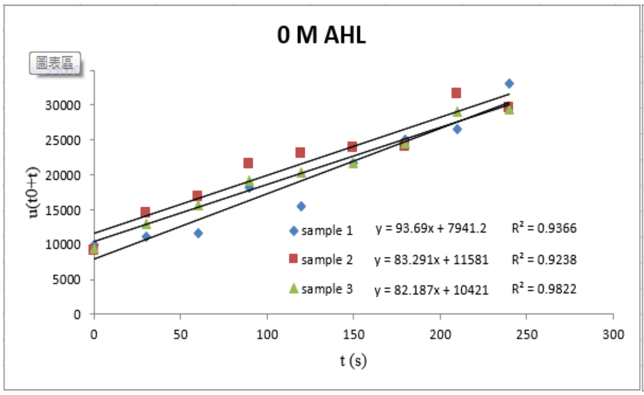

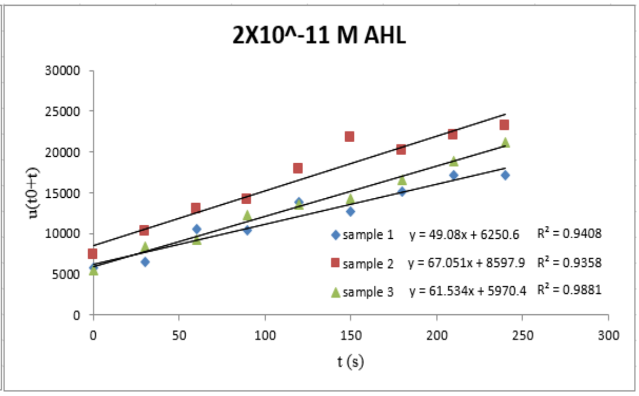

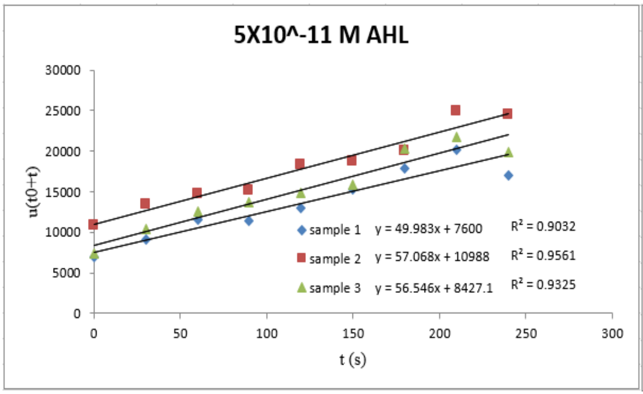

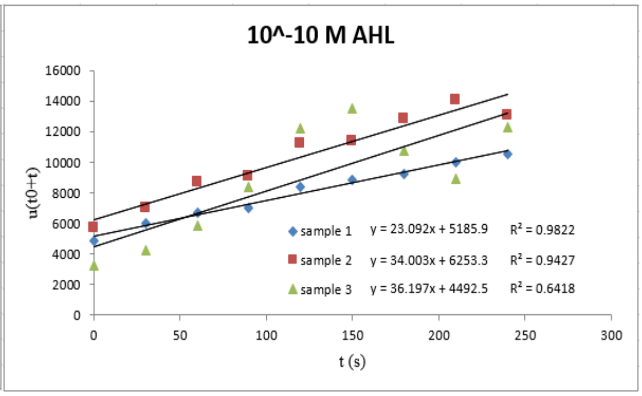

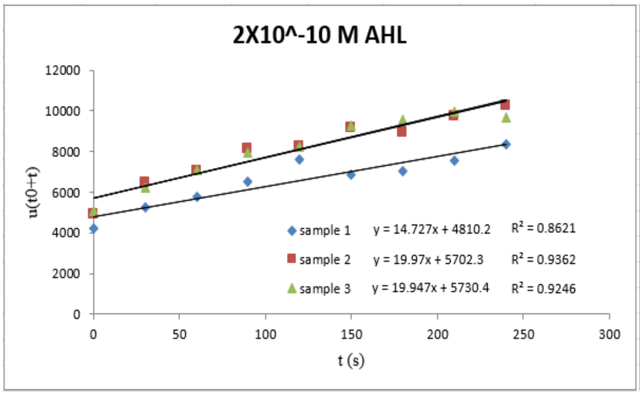

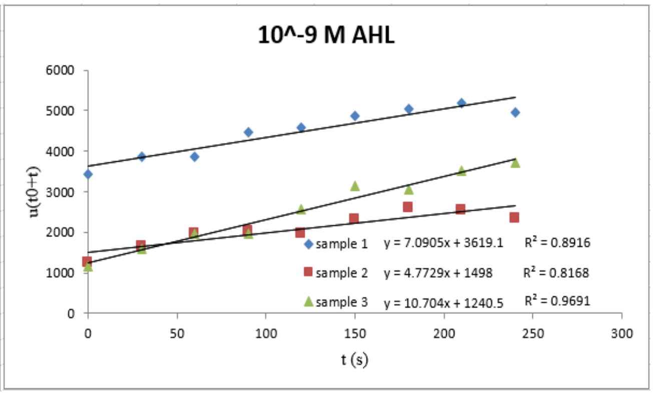

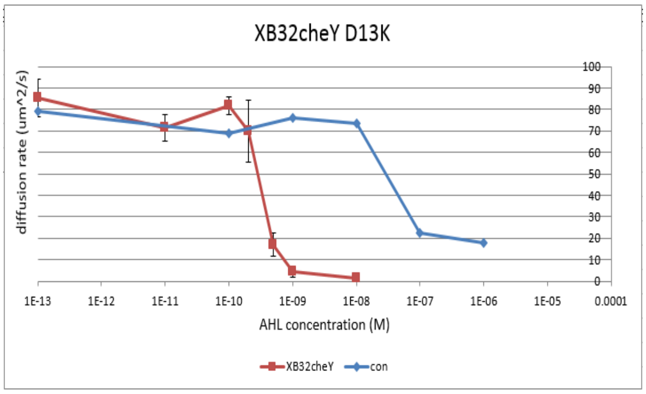

(k) Standard circuit + “B14B32CheY D13K” component

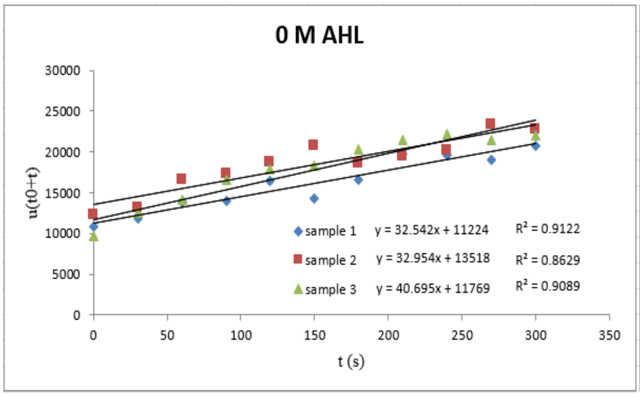

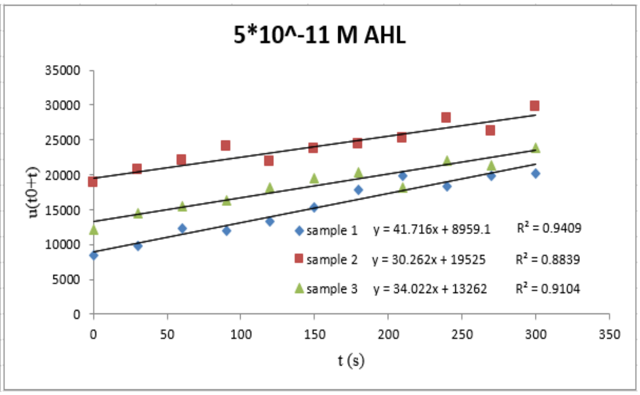

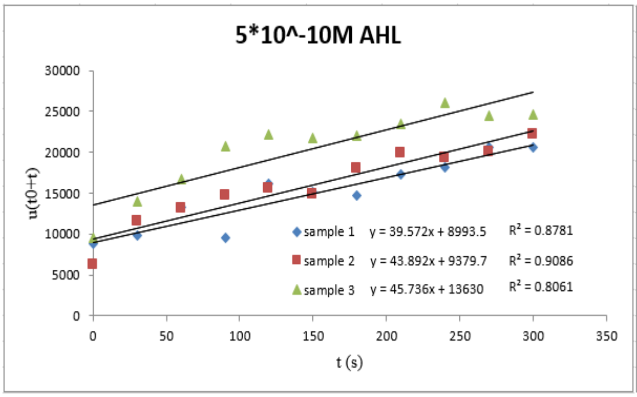

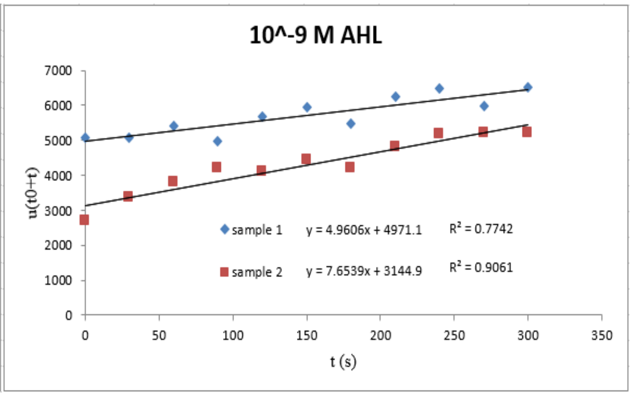

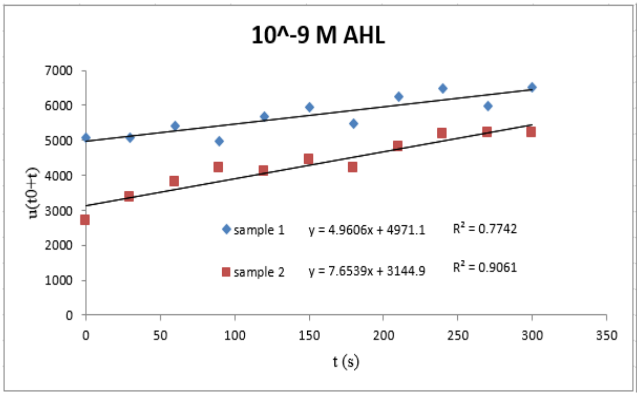

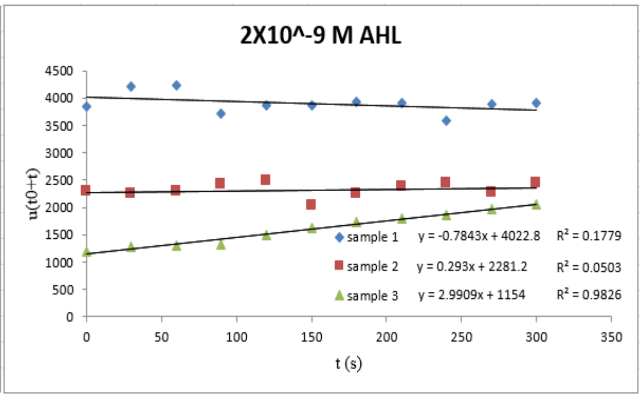

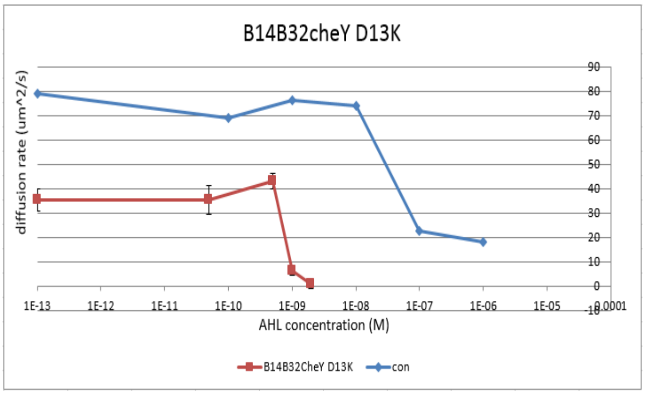

Fig B. Identification of the parameters in Eq. (5) of each brake component.

For each component, the parameters *a* and *b* are identified from the experimental data by genetic algorithm. The results are shown in the figure. Red lines indicate the identified results, whereas blue stars are the experimental data.

“con”

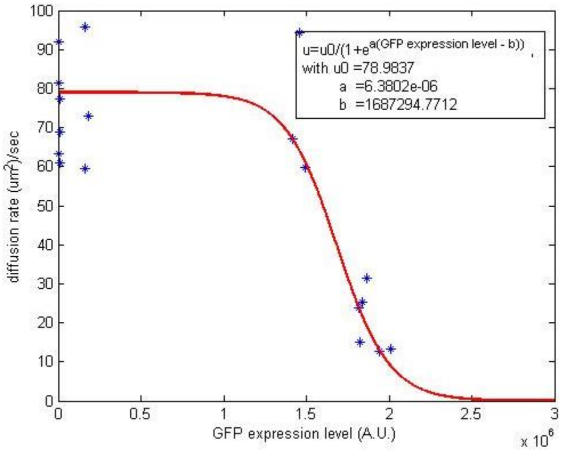

“XB31”

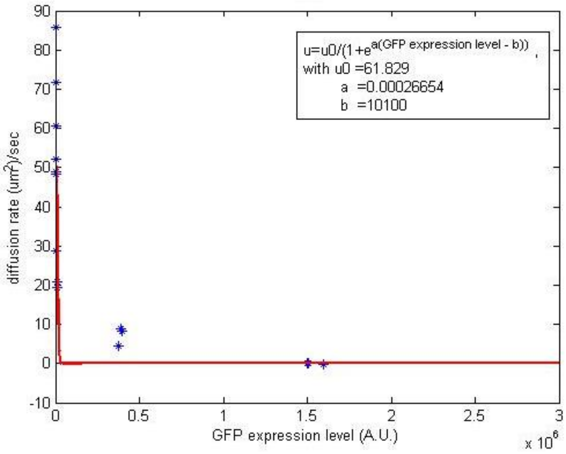

“XB32”

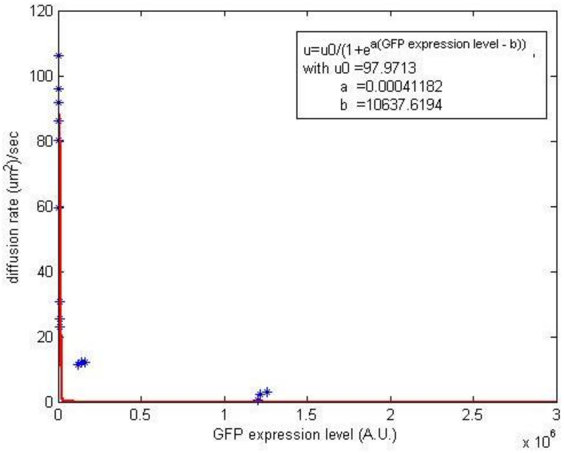

“B14B31”

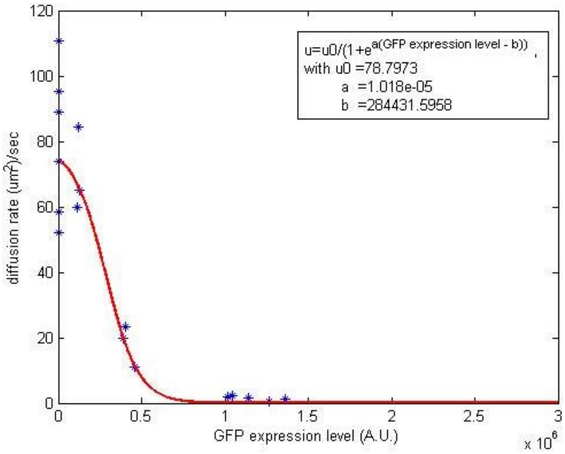

“B14B32”

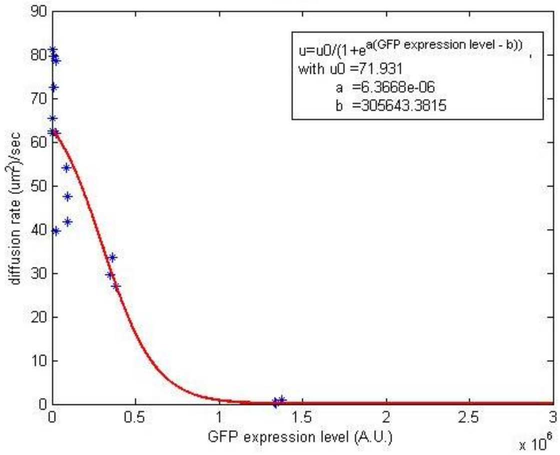

“B15B31”

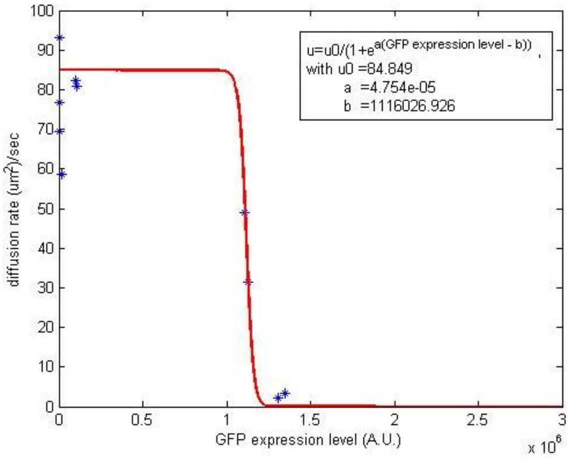

“B15B32”

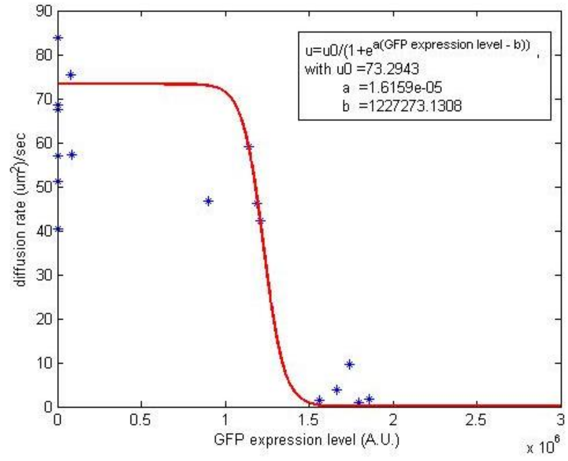

“XB32CheY”

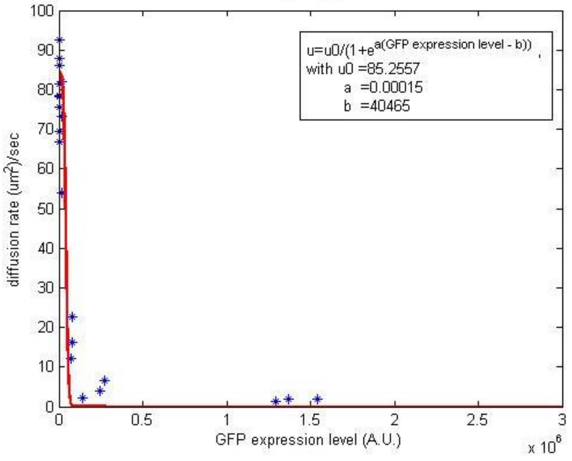

“B14B32CheY”

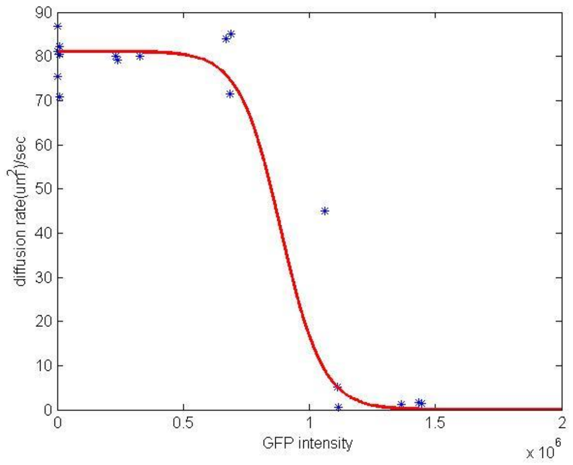

“XB32CheYD13K”

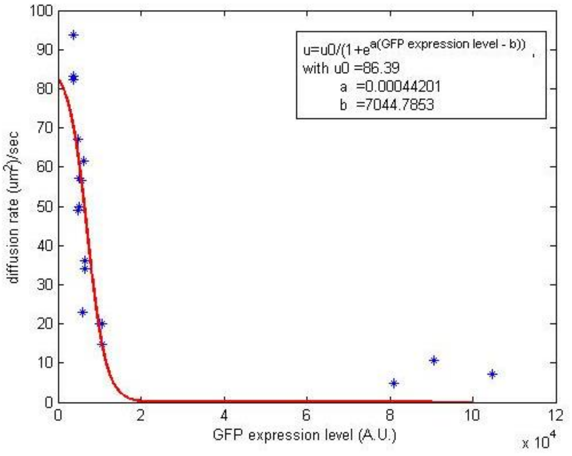

“B14B32CheYD13K”

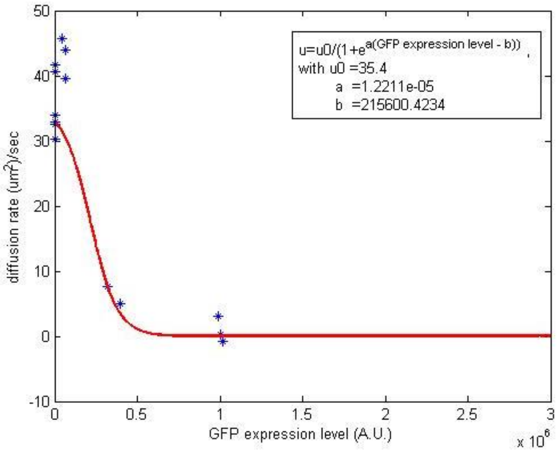

Fig C. GFP expression levels of each brake component.

Figure (a) is the GFP expression level of the standard circuit with the control component "con" at different AHL concentrations. In (b), the results of all components are summarized in four figures classified by the added AHL concentration.

(a)

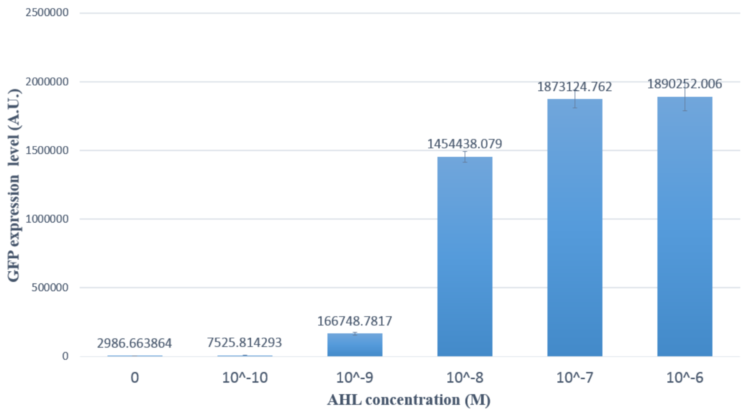

(b)

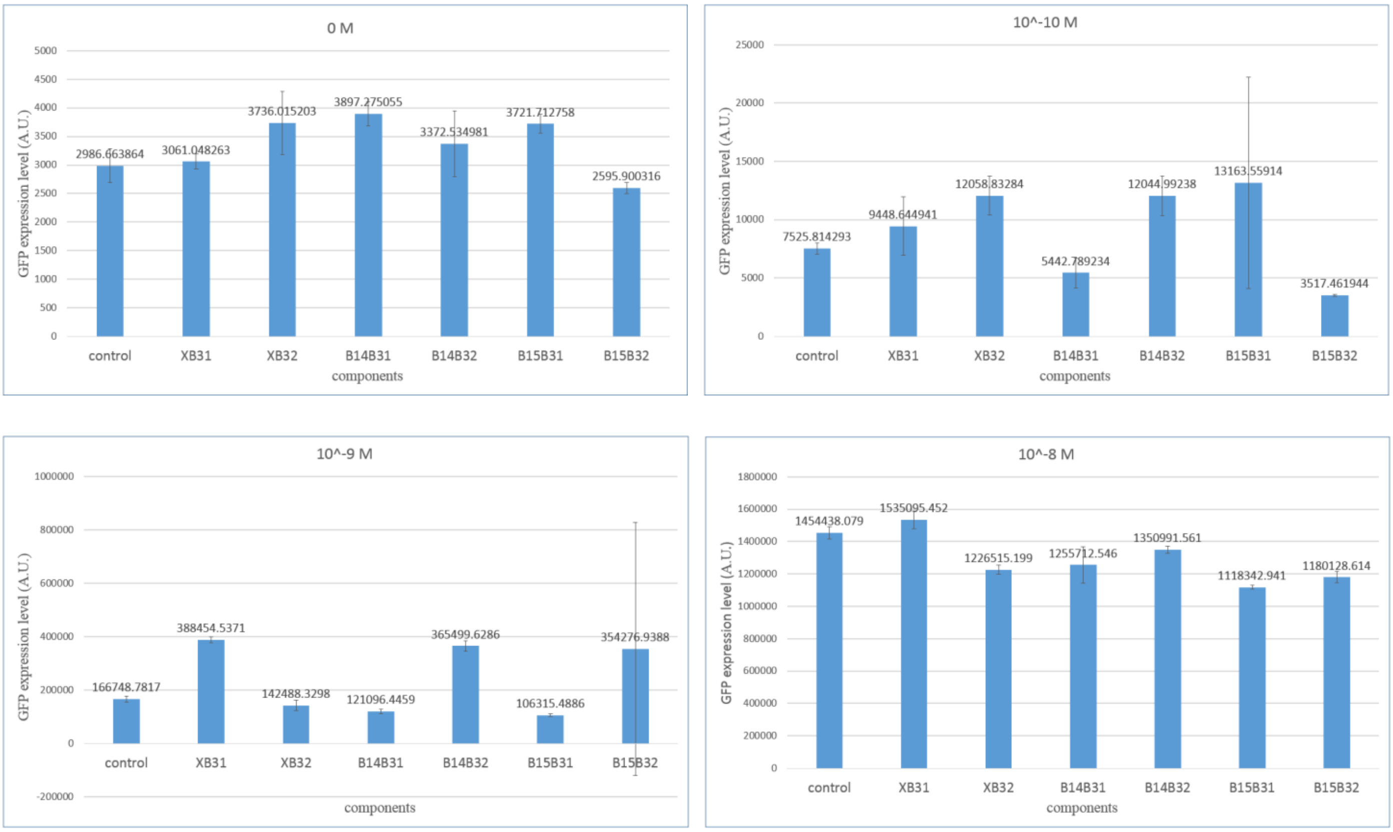

Fig D. Selecting individuals with high moving ability on semi-solid agar by swarm assays.

Figure (a) shows the results of the swarm assay without bacterial selection. By collecting the bacteria from the peripheral of the colony, individuals with higher moving ability on semi-solid agar plate are obtained. Figure (b) is the results of the swarm assays using the chosen bacteria.

(a)

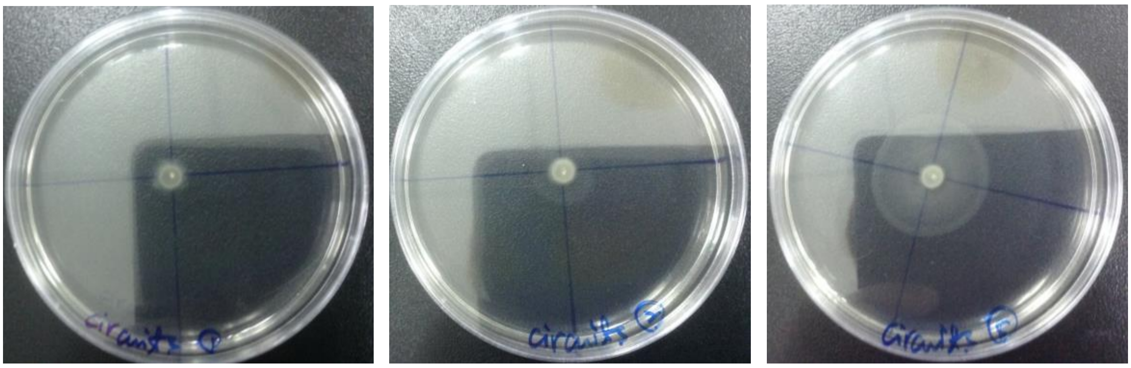

(b)

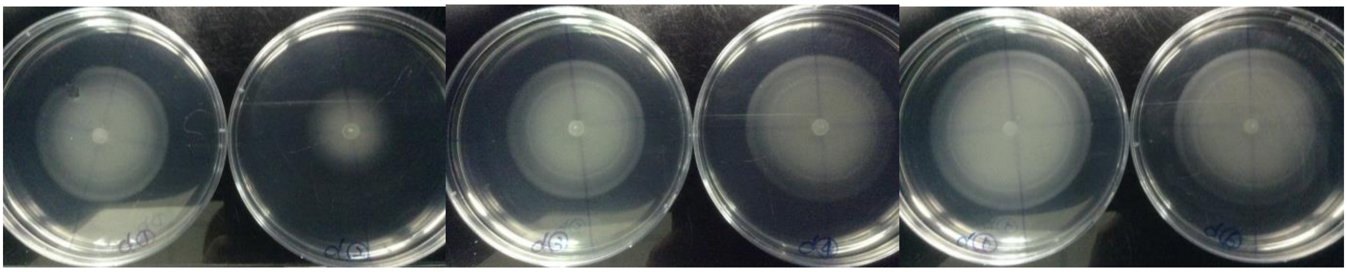

C. Materials and Methods

a. Strains and growth media

Escherichia coli strain RP437 was used as a wild type in this study. RP5232 is a derivative of RP437, which shows a deletion of the CheY gene. Both RP437 and RP5232 were kindly provided by J. S. Parkinson. *Escherichia coli* strain MG1655 was used as the source of some genes used in this study. Details of these *E. coli* strains are listed as follows:

MG1655 F- lambda-ilvG-rfb-50 rph-1

RP437 thr(Am)-1 leuB6 his-4 metF(Am)159 eda-50 rpsL136 [thi-1 ara-14 lacY1 mtl-1 xyl-5 tonA31 tsx-78]

RP5232 (cheY) Δm60-21 thr(Am)-1 leuB6 his-4 metF(Am)159 eda-50 rpsL136 [thi-1 ara-14 lacY1 mtl-1 xyl-5 tonA31 tsx-78]

Two mediums, Luria-Bertani broth (LB) and M9 minimal medium, were used for cultivation of *E. coli* in this study. LB was prepared by dissolving 20 g LB powder (BD) in 1 L water supplemented with 2 mM kanamycin. M9 minimal medium was prepared by dissolving 11.28 g M9 minimal salt 5X (BD) and 2 g casamino acid (BD) powder in 1 L water supplemented with 2 mM kanamycin, 0.2% glucose, 2 uM thiamine HCl, and 2 mM M magnesium sulfate.

b. Genetic circuit construction

All circuit constructions were assembled following the Biobrick assembly method (http://parts.igem.org/Help:Assembly/3A_Assembly) and most of the functional parts were obtained from the Registry of Standard Biological Parts (http://parts.igem.org). The Biobrick sequences including promoters, ribosome binding sites, and terminators we used are listed in Table B. The CheY gene was obtained from *E. coli* strain MG1544 genomic DNA. Two mutants were modified by PCR. All the primers are listed in Table A. The Biobrick Vector pSB1K3, a high copy number plasmid, was used to characterize all the

genetic circuits in this study.

c. Swarm assays

Two mediums, LB and M9, were used to prepare the 0.25% semi-solid agar plate. Plates made by LB medium were used for simply verification of the basic circuit, while plates made by the M9 medium were used to compare brake components.

For the assay with its plate made by LB medium, *E. coli* (MG1655) were cultivated in LB medium at 37℃ overnight and diluted to O.D. 0.05 before being dropped on the semi-solid LB agar plate. After being cultivated for 16 hours, one microliter of 10-3 M, 10-4 M, or 0 M AHL was dropped beside the *E. coli* colonies. The colonies were then cultivated overnight and observed.

For the assay with its plate made by M9 medium, *E. coli* (RP437) were cultivated in M9 medium at 30℃ overnight and diluted (1:100) to fresh M9 minimal medium. After being cultivated for 4 hours, the medium was centrifuged at 3000 rpm for 5 minutes. The pellet was re-suspended in fresh M9 minimal medium to an OD around 0.2 and dropped on the semi-solid M9 agar plate. Simultaneously, 1.5 microliter of specific concentration of AHL was dropped 4 centimeter away from the bacteria as shown in Figure 4. The colonies were cultivated for 20 hours before being observed.

Notably, the bacteria used in the swarm assay needed to be selected previously owing to a greater variability observed between individuals shown in Supplementary Figure D(a). By picking the bacteria from the periphery of the colonies, individuals with better moving ability on the agar were obtained. As shown in Supplementary Figure D(b), the selected individuals not only showed better movement ability on the agar, but also showed little variability among each other. Further, by testing the GFP expression level in response to different AHL concentrations, we demonstrate that there was no significant difference in circuit performance between individuals before and after the screening (data not shown).

As a result, *E. coli* was chosen previously in all the swarm assays.

d. Fabrication of the microchannel

The devices were fabricated using standard soft-lithography techniques. First, negative photoresist (SU-8-2050) of 50 um depth was laid on a 3-inch silicon wafer by spin coating at 3000 rpm twice (8 sec and 45 sec). The wafer was baked twice, at 65 ℃ for 3 minutes and at 95 ℃ for 6 minutes. The wafer was then covered with the mask, a transparency film with the pattern shown in Figure 5(a), and exposed to ultraviolet light using the mask aligner (Karl sussMJB3) for 65 seconds to polymerize the exposed region of photoresist. The wafer with molded pattern was then baked twice, 65 ℃ for 1 minute and 95 ℃ for 8 minutes, and washed by the developer solution SU-8 for 6 minutes. Unexposed photoresist was washed away, which gave an opposite pattern of Figure 5(a) with embossed channels.

Polydimethylsiloxane (PDMS, RTV-615, Momentive, Inc., solution A: solution B=10:1, degassed for 1 hour in a vacuum) was laid on the wafer and hardened by baking at 80℃ for 1 hour to produce a replica with concave channels. A thin layer of silane was laid in advance on the wafer by vaporization to assist the separation of the wafer and the hardened PDMS to avoid the wafer cleaving when peeling from the PDMS. Before sealing the PDMS against a glass microscope slide, holes were poked at the inlets and outlets positions for tubing. Finally, both PDMS and the glass microscope slide were put in the plasma cleaner (Harrick Plasma Cleaner PDC-32, 18W, 1 torr) for 45 seconds and combined together, which formed a covalent bond that sealed both of their surfaces.

e. Diffusion experiment procedure and GFP expression assay

E. coli was cultivated in M9 minimal medium at 30 ℃ on an orbital shaker (250 rpm) overnight and diluted (1:100) into a M9 minimal medium containing different concentrations of chemicals of interest. After being cultivated for 4 hours, the medium was

centrifuged at 3000 rpm for 5 minutes. The pellet was re-suspended in chemical-added M9 minimal medium to an OD around 0.15 to 0.3 (depending on the fluorescent expression level, the weaker the fluorescent presented, the higher the density needed). *E. coli* was then cultivated for 20 minutes again to rewarm the medium and reduce the influence of centrifugation. Just before and after the diffusing rate experiment, the OD and GFP expression levels of the *E. coli* population were measured by an Elisa Reader (BioTek, Synergy H1 Multi-Mode Reader; excitation: 490 nm; emission: 530 nm; gain 50 and 100).

Before the measurement, channels of the microfluidic device were soaked in M9 minimal medium containing 10 mg/mL bovine serum albumin for 30 minutes, which can reduce the adhering of *E. coli* to PDMS during the experiment. The channels were then washed by chemical-added M9 minimal medium to set up the experimental environment. A M9 minimal medium with added chemicals and *E. coli* were pushed into the channel from inlet A and B respectively by a pressure of 4 psi controlled by a computer-controlled gas valve. As the pressure shut down, the flow stopped in seconds. Bacteria were observed by a fluorescent microscope using 10× objective. Images were taken (ANDOR Technology, Zyla sCMDS, 2560 × 2160 pixels, 2 bytes per pixel) for every 30 seconds in 5 minutes. Each image contained 25 continuous frames whose exposure is 200 milliseconds. For every concentration, three repeats of *E. coli* from different colonies were tested in 45 minutes.

f. Data acquisition and processing

Frames at each time point were filtered to remove salt and pepper noise from the frames. To obtain bacterial trajectories, each frame was subtracted from the following one to remove the background and obtain a cleaner image. The distribution of the trajectories was then calculated by counting the pixel numbers at each region, which was at different distances from the center axis. Each region was parallel to the center axis and covered a 50-pixel-wide area. For each sample, the center axis was calculated in advance from the

first frame of the first time point. The distribution line B(x,t) was fitted to Eq. (4) using gene algorism for 1000 runs, which yielded the value of $\mu(t + t_0)$. Diffusion rate μ was acquired by handling values of $\mu(t + t_0)$ over time by linear regression.
